# Grafting nanometer metal/oxide interface towards enhanced low-temperature acetylene semi-hydrogenation

**DOI:** 10.1038/s41467-021-25984-8

**Published:** 2021-10-01

**Authors:** Shihui Zou, Baohui Lou, Kunran Yang, Wentao Yuan, Chongzhi Zhu, Yihan Zhu, Yonghua Du, Linfang Lu, Juanjuan Liu, Weixin Huang, Bo Yang, Zhongmiao Gong, Yi Cui, Yong Wang, Lu Ma, Jingyuan Ma, Zheng Jiang, Liping Xiao, Jie Fan

**Affiliations:** 1 Key Lab of Applied Chemistry of Zhejiang Province, Department of Chemistry, Zhejiang University, 310027 Hangzhou, China; 2 School of Physical Science and Technology, ShanghaiTech University, 201210 Shanghai, China; 3 School of Materials Science and Engineering, Zhejiang University, 310027 Hangzhou, China; 4 Center for Electron Microscopy, State Key Laboratory Breeding Base of Green Chemistry Synthesis Technology and College of Chemical Engineering, Zhejiang University of Technology, 310014 Hangzhou, China; 5 Institute of Chemical and Engineering Sciences, A*STAR, 627833 Singapore, Singapore; 6 National Synchrotron Light Source II, Brookhaven National Laboratory, Upton, NY 11973 USA; 7 College of Materials & Environmental Engineering, Hangzhou Dianzi University, 310036 Hangzhou, China; 8 Department of Chemical Physics, University of Science and Technology of China, 230026 Hefei, China; 9 Vacuum Interconnected Nanotech Workstation, Suzhou Institute of Nano-Tech and Nano-Bionics, Chinese Academy of Sciences, 215123 Suzhou, China; 10 Shanghai Synchrotron Radiation Facility, Shanghai Institute of Applied Physics Chinese Academy of Sciences, 201800 Shanghai, China

**Keywords:** Catalyst synthesis, Heterogeneous catalysis, Chemical engineering

## Abstract

Metal/oxide interface is of fundamental significance to heterogeneous catalysis because the seemingly “inert” oxide support can modulate the morphology, atomic and electronic structures of the metal catalyst through the interface. The interfacial effects are well studied over a bulk oxide support but remain elusive for nanometer-sized systems like clusters, arising from the challenges associated with chemical synthesis and structural elucidation of such hybrid clusters. We hereby demonstrate the essential catalytic roles of a nanometer metal/oxide interface constructed by a hybrid Pd/Bi_2_O_3_ cluster ensemble, which is fabricated by a facile stepwise photochemical method. The Pd/Bi_2_O_3_ cluster, of which the hybrid structure is elucidated by combined electron microscopy and microanalysis, features a small Pd-Pd coordination number and more importantly a Pd-Bi spatial correlation ascribed to the heterografting between Pd and Bi terminated Bi_2_O_3_ clusters. The intra-cluster electron transfer towards Pd across the as-formed nanometer metal/oxide interface significantly weakens the ethylene adsorption without compromising the hydrogen activation. As a result, a 91% selectivity of ethylene and 90% conversion of acetylene can be achieved in a front-end hydrogenation process with a temperature as low as 44 °C.

## Abstract (web summary)

Metal/oxide interface is of fundamental significance to heterogeneous catalysis. Here, the authors construct a nanometer Pd/Bi_2_O_3_ interface by grafting Pd clusters onto Bi_2_O_3_ clusters and demonstrate its essential roles in the low-temperature semi-hydrogenation of acetylene.

## Introduction

Metal/oxide interface is of great fundamental and practical interest to heterogeneous catalysis, because it raises essential questions regarding the strong metal–support interaction^1,2^ and plays a pivotal role in several catalytic processes^3,4^. From a structural perspective, a metal/oxide interface is constructed by components that significantly differ from each other in terms of chemical compositions, bonding characters, lattice parameters, and electric and mechanical properties^5,6^, of which the adhesion structure and chemistry turn out to be a compelling research topic, while from a functional perspective, the chemical bonding and associated charge transfer^7^ at the metal/oxide interface allow the modulations of morphology, size, and electronic structures of metals to optimize the bonding strength of reaction intermediates for better catalytic performances^3^. During the past few decades, considerable progress has been achieved in the structural elucidation and tuning of well-defined metal/oxide interfaces that usually adopt a bulk oxide support to facilitate the nucleation, adsorption, or deposition of metals^8^. It is expected that a nanometer metal/oxide interface, perhaps formed by heterografting between metal and oxide clusters, would reinforce the structural and electronic effect to achieve better catalytic performance. However, due to the great challenges in the chemical synthesis and structural elucidation of such hybrid clusters, there are limited insights into the nanometer metal/oxide interface.

As a representative reaction where oxides supported metal catalysts are frequently used, the selective hydrogenation of acetylene to ethylene is subjected to an inherent trade-off between two essential requirements for both high catalytic activity and selectivity: the facile activation of hydrogen and the weak binding of ethylene^9^. Despite the significant progress achieved by Pd-based catalysts^10^, a simultaneous optimization of these two parameters is still challenging, especially in the front-end process where H_2_ and C_2_H_4_ are in large excess. To reach this goal requires a sophisticated tuning of the geometric and electronic structure of Pd, which motivates people to engineer the metal/oxide interface. In most Pd/oxide catalysts, only Pd nanoparticles or isolated Pd atoms are loaded. Unfortunately, Pd nanoparticles are efficient to activate hydrogen at low temperatures, but their strong binding with ethylene favors sequential hydrogenation of ethylene to ethane^10,11^. Isolated Pd site catalysts including Pd single-atom catalysts^12–15^ and Pd-based intermetallic compounds^16–22^ feature weak π-bonding with ethylene and thus good selectivity in the acetylene hydrogenation reaction, but their concomitant weakened hydrogen activation requires a relatively high reaction temperature (>100 °C) to achieve high conversion of acetylene, which potentially leads to a safety concern in the reactor beds^21^. Decreasing the size of oxide supports to nanocluster scale would remarkably change their coordination number (CN)^23^, surface termination^24^, and *d*-band character^25^, which allow a strong chemical and electronic interaction with Pd to continuously regulate the size and electronic structure of Pd. Among them, ligand-free Pd clusters stabilized by a nanometer metal/oxide interface are expected to bridge the size and performance gaps between Pd nanoparticles and single atoms with maximized interfacial effects.

Herein we propose a facile stepwise photochemical strategy to fabricate hybrid Pd/Bi_2_O_3_ clusters where the Pd clusters are stabilized by the ~1 nm ordered Bi_2_O_3_ cluster through the nanometer interfaces. These hybrid clusters are well and stably dispersed on TiO_2_ substrate with a high Pd loading up to 2.3 wt.%. They exhibit a low Pd–Pd CN of 4.7 and more importantly a Pd–Bi spatial correlation ascribed to the heterografting between Pd and Bi-terminated Bi_2_O_3_ clusters. Interestingly, these hybrid clusters feature an intra-cluster electron transfer toward Pd and result in a deeper *d*-band center compared with other Pd metals, which enables much weaker ethylene binding without compromising the hydrogen activation activity. As a result, a 90% conversion of acetylene together with a 91% selectivity to ethylene is achieved in excess of ethylene and at a temperature as low as 44 °C.

## Results and discussion

### Synthesis and structural characterizations

**Fig. 1:**
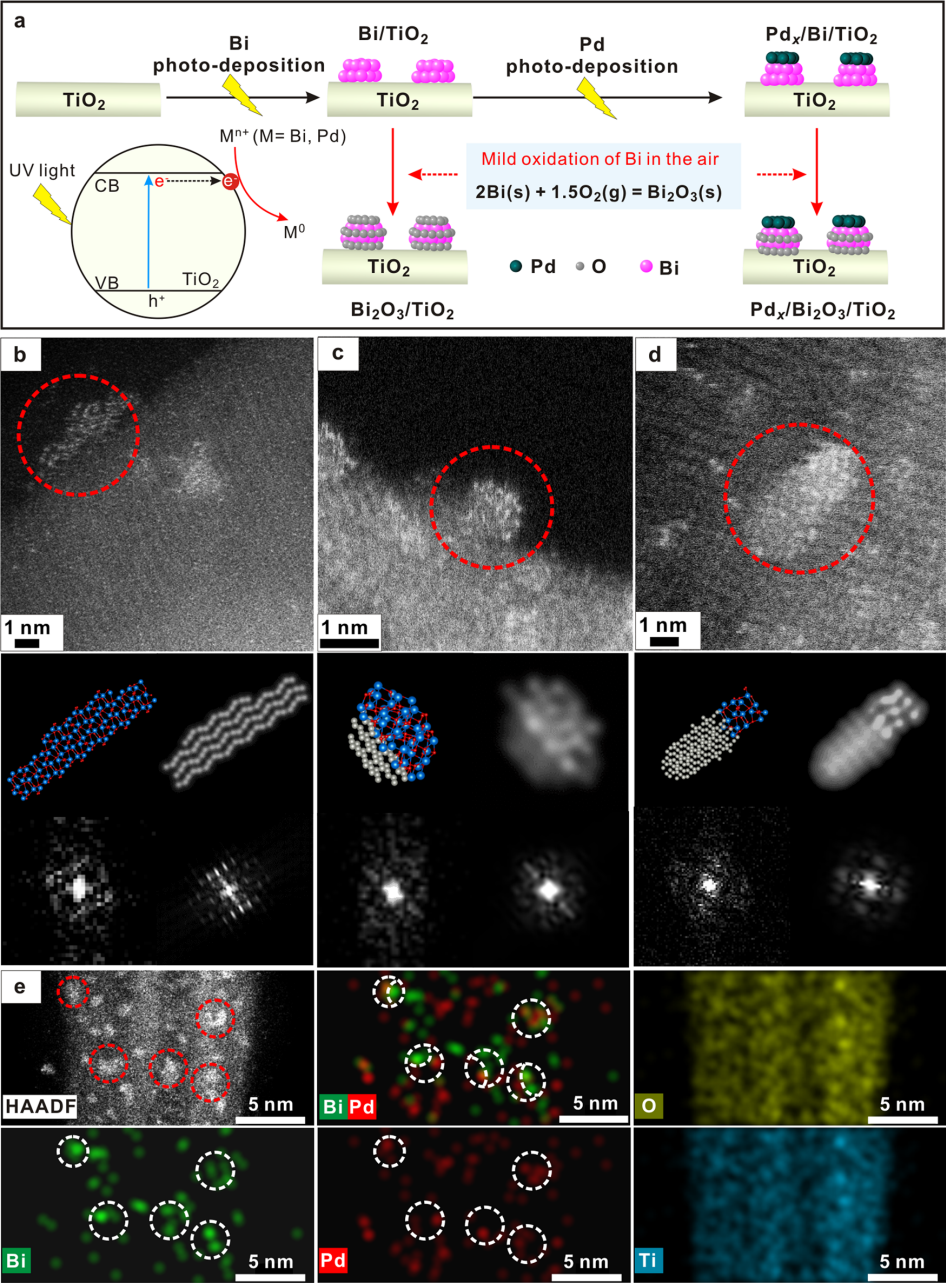
Microstructure of Pd_1.0_/Bi_2_O_3_/TiO_2_. **a** Schematic illustration of the synthetic procedures. **b**–**d** STEM images of Bi_2_O_3_/TiO_2_ (**b**) and Pd_1.0_/Bi_2_O_3_/TiO_2_ (**c**, **d**). The insets from upper to lower and left to right: HRSTEM images, projected structural models, simulated ADF-STEM images, FFTs from dashed circular regions in HRSTEM images, and FFTs from simulated ADF-STEM images, respectively. **e** Elemental mapping of Pd_1.0_/Bi_2_O_3_/TiO_2_.

Pd_1.0_/Bi_2_O_3_/TiO_2_ catalysts were prepared by a stepwise photo-deposition of Bi and Pd on TiO_2_ with a molar ratio of Bi/Pd kept at 1.0 (please see details in “Methods”). In brief, Bi^3+^ was reduced by the photogenerated electrons to produce Bi^0^ clusters on TiO_2_. Subsequently, Pd was deposited onto Bi/TiO_2_ to form Pd/Bi/TiO_2_. When Bi/TiO_2_ and Pd/Bi/TiO_2_ were exposed to the air, Bi was spontaneously oxidized into Bi_2_O_3_ because the Gibbs free energy of the reaction is minus^26^. The synthetic procedure is schematically illustrated in Fig. 1a. Inductively coupled plasma–atomic emission spectroscopy (ICP-AES) suggests that the mass loading of Bi and Pd are 4.9 wt.% and 2.3 wt.%, respectively, close to the feeding ratio. Our previous study has proved that TiO_2_ has a strong interaction with Bi^3+^, characterized by an unprecedented 1.5 eV upshift of Ti 2*p*^27^. Such interaction ensures the formation of highly dispersed Bi on TiO_2_ during the following reduction of Bi^3+ ^^28^. As shown in Supplementary Fig. 1a, b, Bi species are uniformly dispersed on TiO_2_ as ~1 nm clusters with a Bi loading up to 5 wt.%. Interestingly, close inspection of individual Bi cluster on the TiO_2_ support by using aberration-corrected annular dark-field scanning transmission electron microscopy (ADF-STEM) indicates that the Bi cluster has an ordered α-Bi_2_O_3_ structure with a highly distorted lattice, which exhibits relatively weak diffuse peaks in the fast Fourier transform (FFT; Fig. 1b). This is consistent with the literature result^26^, which indicates that the mild oxidation of Bi^0^ in the air is thermodynamically spontaneous and usually forms monoclinic α-Bi_2_O_3_ (monoclinic, *P*2_1_/c(14)). In addition to the intrinsic lattice distortion that blurs the image contrast, the beam-induced structural dynamics of the highly beam-sensitive Bi_2_O_3_ clusters may further introduce image blurring, mainly arising from the remarkable knock-on displacements and radiolysis. Despite these beam-induced effects, careful inspection on these ADF-STEM images allows the identification of local contrasts that closely resemble the projected structures and simulated ADF-STEM image of Bi_2_O_3_ along [100] (Fig. 1b). Specifically, the structural projection and simulated ADF-STEM image of ordered Bi_2_O_3_ feature a wave-shaped arrangement of Bi atomic columns from the [100] projection, while the experimental ADF-STEM image exhibits a similar contrast but with more lattice distortions (Fig. 1b). Accordingly, the FFT pattern of the experimental image exhibits rather diffuse spots compared with those in the FFT pattern of simulated image.

**Fig. 2:**
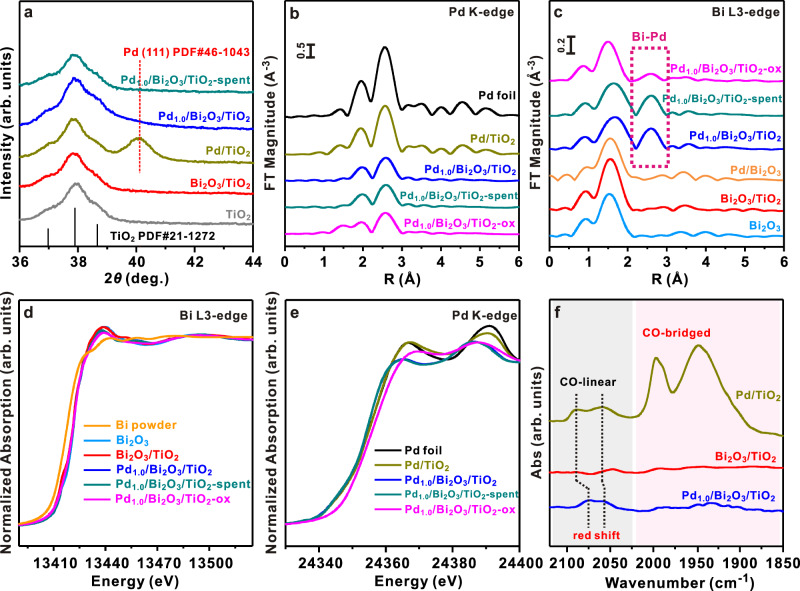
Characterization of Pd_1.0_/Bi_2_O_3_/TiO_2_. **a** XRD patterns of TiO_2_, Pd/TiO_2_, Bi_2_O_3_/TiO_2_, and Pd_1.0_/Bi_2_O_3_/TiO_2_; **b** Fourier transform spectra of Pd *K*-edge EXAFS for Pd/TiO_2_, Pd_1.0_/Bi_2_O_3_/TiO_2_, and oxidized Pd_1.0_/Bi_2_O_3_/TiO_2_ (Pd_1.0_/Bi_2_O_3_/TiO_2_-ox); **c** Fourier transform spectra of Bi L3-edge EXAFS; **d** Bi L3-edge XANES spectra for Bi_2_O_3_/TiO_2_, Pd_1.0_/Bi_2_O_3_/TiO_2_, and Pd_1.0_/Bi_2_O_3_/TiO_2_-ox. Bi and Bi_2_O_3_ powder were used as references. **e** Pd *K*-edge XANES spectra for Pd/TiO_2_, Pd_1.0_/Bi_2_O_3_/TiO_2_, and Pd_1.0_/Bi_2_O_3_/TiO_2_-ox. Pd foil was used as a reference. **f** CO-adsorbed FT-IR spectra for various samples. Source data are provided in a Source data file.

Followed by the secondary deposition of Pd, the clusters of Bi_2_O_3_ intergrown with Pd particles can be found according to the low-magnification ADF-STEM images (Supplementary Fig. 1). Besides, Pd_1.0_/Bi_2_O_3_/TiO_2_ exhibits no signals for Pd in the X-ray diffraction (XRD) patterns (Fig. 2a) while photo-deposited Pd/TiO_2_ with an identical Pd loading (2.5 wt.%) shows a characteristic Pd(111) diffraction peak^29^ at 40.1^o^, suggesting that pre-deposited Bi_2_O_3_ clusters assist in the high dispersion of Pd species in Pd_1.0_/Bi_2_O_3_/TiO_2_. According to the literatures^30,31^, pre-deposited component with high work function can serve as a sink of photo-induced electrons, which preferentially reduce the second metal on the surface of the pre-deposited metal. By fixing the loading of Bi at 5.0 wt.%, we can also fabricate Pd_0.5_/Bi_2_O_3_/TiO_2_ (Supplementary Fig. 2), Pd_0.2_/Bi_2_O_3_/TiO_2_, and Pd_0.1_/Bi_2_O_3_/TiO_2_ with high dispersion of Pd (Pd_*x*_/Bi_2_O_3_/TiO_2_, *x* represents Pd-to-Bi molar ratio). In contrast, when we increase Pd/Bi ratio to 3.0, the excess Pd also deposits directly on TiO_2_. As a result, Pd nanoparticles are obtained (~6.7 nm, Supplementary Fig. 3). Taking all above results together, Pd species are most likely deposited on the existing Bi_2_O_3_ clusters although the discrimination between Pd and Bi_2_O_3_ components is not straightforward by STEM imaging due to their small size and irradiation vulnerability. The nanoscopic elemental distribution can be mapped by a Super-X energy dispersive X-ray spectroscope (EDS) system with superior sensitivity. As shown in Fig. 1e and Supplementary Fig. 4, elemental mappings of Pd and Bi components suggest that their spatial distribution in most cases are correlated. In other words, these clusters are bicomponent with segregated Pd- and Bi-containing hemi-clusters, which unambiguously confirm the Pd-grafted Bi_2_O_3_ hybrid cluster structure. Careful inspection on several representative ADF-STEM images of Pd_1.0_/Bi_2_O_3_/TiO_2_ (Fig. 1c, d and Supplementary Fig. 5) allows the discrimination of Pd- and Bi-containing hemi-clusters from their distinct contrasts for adjacent clusters. The hemi-clusters featuring less bright contrast (marked by circle) are directly attached to brighter ones assigned to Bi_2_O_3_ hemi-clusters, further confirming that Pd is grafted onto the surface of Bi_2_O_3_ clusters. Notably, most such Pd clusters are identified to bond and hybridize with Bi_2_O_3_ clusters without any fixed orientation relationship or facet preference, probably due to the ultra-small size and large strain of the clusters. Two representative atomic-resolution ADF-STEM images of Pd-Bi_2_O_3_ nanoclusters with well-resolved Pd- and Bi-containing hemi-clusters are shown in Fig. 1c, d, of which the image contrasts and FFT patterns closely resemble the simulated ones of artificially constructed hybrid cluster models with different orientation relationships between two types of hemi-clusters made of Pd and α-Bi_2_O_3_ structures, respectively. The above observations unambiguously validate the proposed Pd–Bi_2_O_3_ hybrid structural model. Quite rarely, such Pd clusters are observed to directly nucleate onto the TiO_2_ substrate.

To confirm the coordination environment of Pd clusters, X-ray absorption fine structure (XAFS) of Pd *K*-edge and Bi L3-edge were performed, as shown in Fig. 2b–e. In the Fourier transformed extended X-ray absorption fine structure (FT-EXAFS) data of Bi L3-edge, it is important to note that a new coordination peak in *R*-space at about 2.6 Å is observed on Pd_1.0_/Bi_2_O_3_/TiO_2_ (Fig. 2c), but it is totally absent on other samples containing Bi_2_O_3_ (i.e., Bi_2_O_3_ and Bi_2_O_3_/TiO_2_). Considering that the only difference between Pd_1.0_/Bi_2_O_3_/TiO_2_ and Bi_2_O_3_/TiO_2_ is the secondary deposition of 2.3 wt.% Pd on Bi_2_O_3_/TiO_2_, the peak at 2.6 Å should be contributed by the Bi–Pd interaction. Interestingly, the peak can be well fitted by a single Bi–Pd shell (Supplementary Fig. 6 and Supplementary Table 1), suggesting it is a characteristic peak of Bi–Pd coordination. To further prove this idea, the Pd_1.0_/Bi_2_O_3_/TiO_2_ is mildly treated under air at 150 °C (denoted as Pd_1.0_/Bi_2_O_3_/TiO_2_-ox) to oxidize Pd species while maintaining the structural integrity of Bi_2_O_3_ clusters^32^. In Fig. 2e, Pd_1.0_/Bi_2_O_3_/TiO_2_-ox shows clearly blue shift of the absorption edge position comparing with Pd_1.0_/Bi_2_O_3_/TiO_2_, indicating the oxidation of Pd after the treatment. Meanwhile, the peak at ~2.6 Å of Bi L3-edge EXAFS diminishes significantly upon oxidation of Pd_1.0_/Bi_2_O_3_/TiO_2_ (Fig. 2c). The fitting results reveal that the Bi–Pd CN is decreased from 2.9 for Pd_1.0_/Bi_2_O_3_/TiO_2_ to 1.1 for Pd_1.0_/Bi_2_O_3_/TiO_2_-ox (Supplementary Table 1). Accordingly, this experiment identifies that the peak at 2.6 Å is from Bi–Pd bond and in turn proves the Pd–Bi pairs across the interfaces of Pd/Bi_2_O_3_ hybrid clusters observed by ADF-STEM in Fig. 1c, d. The Bi–Pd interaction eventually changes the coordination environment of Pd in Pd_1.0_/Bi_2_O_3_/TiO_2_. As compared to Pd foil and Pd/TiO_2_, the significantly weaker and slightly broader coordination peak in the Pd *K*-edge EXAFS of Pd_1.0_/Bi_2_O_3_/TiO_2_ (Fig. 2b) implies a decreased CN of Pd–Pd pairs and distorted structure for the Pd clusters similarly as observed by STEM imaging^33^. The fitting of Pd *K*-edge EAXFS (Supplementary Fig. 7 and Supplementary Table 2) further confirms that the presence of Pd–Bi coordination (CN = 4.6) decreases the Pd–Pd CN from 10.0 for Pd/TiO_2_ to 4.7 for Pd_1.0_/Bi_2_O_3_/TiO_2_, which is consistent with Pd cluster structure observed by ADF-STEM. The Pd–Bi bond length obtained from the fitting of Pd *K* and Bi L3 edge is 2.79 ± 0.03 Å, suggesting the observed spatial correlation of Pd–Bi pairs arises from the direct bonding between Pd- and Bi-terminated clusters instead of the Pd–O–Bi moieties that attain a larger interatomic distance (~3.5 Å). Photo-deposition procedure is critical to ensure the formation of direct Pd–Bi bonding in Pd/Bi_2_O_3_ clusters as depicted in Fig. 1a. Theoretically, Bi_2_O_3_ favors an oxygen termination. Pd supported on Bi_2_O_3_ would be in direct contact with O rather than Bi. We prepared a Pd/Bi_2_O_3_ sample by directly depositing Pd onto commercial Bi_2_O_3_ support. Interestingly, no characteristic peak at ~2.6 Å was observed in the Bi L3 EXAFS of Pd/Bi_2_O_3_, excluding the presence of Pd–Bi bond (Fig. 2c). In this study, photo-deposition procedure ensures the Pd cluster deposited on reduced Bi^0^ clusters and allows the formation of Pd–Bi bonding during the synthesis as depicted in Fig. 1a. The Pd–Bi bond preserves during the mild oxidation of Bi to Bi_2_O_3_ at room temperature (RT) that occurred after the deposition of Pd onto Bi/TiO_2_, as evidenced by the characteristic Bi–Pd peak in Bi L3 EXAFS (Fig. 2c). More interestingly, 36% of this peak preserves even after oxidation in air at a higher temperature of 150 °C (Pd_1.0_/Bi_2_O_3_/TiO_2_-ox). These results clearly indicate the good stability of Pd–Bi bond in Pd_1.0_/Bi_2_O_3_/TiO_2_ and suggest that Pd supported on Bi-terminated Bi_2_O_3_ as the structural model for Pd_1.0_/Bi_2_O_3_/TiO_2_ in the hydrogenation reaction conditions. Taken together, the above-mentioned results unambiguously confirmed that the Pd species are predominantly in the form of nanometer-sized Pd clusters embedded in the Pd/Bi_2_O_3_ hybrid clusters through chemical adhesion.

In order to identify the electronic structure of the as-synthesized Pd/Bi_2_O_3_ hybrid clusters in reaction conditions, in situ X-ray photoelectron spectra (XPS) was collected at 100 °C under H_2_ atmosphere (Supplementary Fig. 8). As shown in Supplementary Fig. 8b, there are symmetric Bi 4*f* peaks at 158.8/164.1 eV, confirming that the majority of Bi are in the form of Bi_2_O_3_^34^. Both Bi_2_O_3_/TiO_2_ and Pd_1.0_/Bi_2_O_3_/TiO_2_ exhibit similar Bi L3-edge structures with Bi_2_O_3_ by inspecting the X-ray absorption near edge structure (XANES), indicating similar valence states among them (Fig. 2d). It is frequently reported that intermetallic compounds (IMC) could be possibly formed by a so-called reactive metal–support interaction^35^. However, according to the density functional theory (DFT) calculations (Supplementary Table 3), the Pd–Bi distance in PdBi IMC ranges from 2.85 to 3.03 Å, which is obviously larger than that of Pd_1.0_Bi/TiO_2_ (2.79 Å, Supplementary Table 2). This result excludes PdBi IMC as the main phase of Pd_1.0_/Bi_2_O_3_/TiO_2_. To better illustrate the difference between Pd–Bi_2_O_3_ nanoclusters and PdBi intermetallic compounds, we synthesized a PdBi IMC (denoted as PdBi/TiO_2_) for comparison (Supplementary Fig. 9). XRD patterns (Supplementary Fig. 9a) of PdBi/TiO_2_ exhibit characteristic peaks at 39.9 and 42.8°, corresponding to (102) and (110) of hexagonal PdBi IMC (sobolevskite, *P*6_3_/mmc(194), *a* = *b* = 4.22 Å, *c* = 5.709 Å, *α* = β = 90°, and *γ* = 120°). Pd–M (M = Pd or Bi) coordination peaks in *R* space of PdBi/TiO_2_ obviously shift (~0.06 Å) from that of Pd_1.0_/Bi_2_O_3_/TiO_2_, confirming that PdBi IMC have longer Pd–Bi distances than Pd–Bi_2_O_3_ nanoclusters (Supplementary Fig. 9b). More importantly, a massive Bi^0^ peak at 156.6/162.0 eV (Supplementary Fig. 9d) is observed in the Bi 4*f* of PdBi/TiO_2_ but is absent in that of Pd_1.0_/Bi_2_O_3_/TiO_2_ (Supplementary Fig. 8b). These results clearly indicate that Pd_1.0_/Bi_2_O_3_/TiO_2_ is composed of Pd–Bi_2_O_3_ hybrid clusters rather than PdBi IMC. It is reasonable because the reaction temperature in this study is too low to reduce Bi_2_O_3_ to Bi, which is a prerequisite for the formation of PdBi IMC. On the other side, the Pd 3*d* patterns of Pd/TiO_2_ and Pd_1.0_/Bi_2_O_3_/TiO_2_ exhibit a slightly asymmetric lineshape (Supplementary Fig. 8a), which is most likely due to the many-body screening response of conduction electrons to the photoemission of a core electron^36^. The predominant Pd 3*d* signals of Pd/TiO_2_ and Pd_1.0_/Bi_2_O_3_/TiO_2_ locate at ~334.9/340.0 eV, which are characteristic of Pd^0^. It is important to note that, due to the differences in the extra-atomic relaxation of metal particles of different sizes, decreasing the particle size of Pd generally upshifts Pd 3*d* to higher binding energy (BE)^37^. In this study, the Pd 3*d* and 3*p* of Pd_1.0_/Bi_2_O_3_/TiO_2_ downshift slightly to lower BE although the particle size of Pd_1.0_/Bi_2_O_3_/TiO_2_ is significantly smaller than that of Pd/TiO_2_ (Supplementary Figs. 8a, c). These results, opposite to the particle-size-induced BE shift, indicate a charge transfer from Bi to Pd. Similar result was also reported in Au/TiO_2_ system^38^.

The more important feature associated with the electronic structure of Pd clusters is observed in the Pd *K*-edge and L3-edge structures as shown in Fig. 2e and Supplementary Fig. 10. Specifically, the Pd *K*-edge XANES profile of Pd/TiO_2_ is very similar to that of Pd foil. In contrast, Pd_1.0_/Bi_2_O_3_/TiO_2_ shows a marked red-shift of the absorption edge energy and decrease in the white line intensity. This indicates the electron-richness of Pd atoms in Pd_1.0_Bi/TiO_2_ compared to those in Pd foil and Pd/TiO_2_, which most likely arises from the Bi_2_O_3_-to-Pd intra-cluster electron transfer. Moreover, Pd_1.0_/Bi_2_O_3_/TiO_2_ shows a much weaker Pd L3 white line intensity at ~3173 eV than Pd/TiO_2_ (Supplementary Fig. 10), which indicates an enhanced *d*-band filling. Such phenomenon is similarly predicted by the Bader charge analysis over Pd_8_ clusters supported by the Bi_2_O_3_ cluster as shown in Supplementary Fig. 11. It is quite surprising to observe such an enhanced *d*-band filling and associated negative shift of *d*-band center away from the Fermi level for nanometer-sized Pd clusters, because the strong “size effect” of most supported metals usually leads to a decreased *d*-band filling and thus positive shift of *d*-band center toward Fermi level for smaller nanoparticles^39^. Actually, this has become a limiting factor for applying supported metal nanoparticles in the selective acetylene hydrogenation reaction due to the strong adsorption of ethylene molecules on electron-deficient metals and results in over-hydrogenation. The metal termination of Bi_2_O_3_ observed here at the Pd–Bi interface of the hybrid cluster could lead to a strong downshift of its surface conduction band. Similar results were also reported in metal–metal interface of Pd/MgO, Pd/ZnO, Ru/MgO, and Au/TiO_2_^38,40–42^. Electrons transferred from terminated magnesium or zinc to adsorbed Pd can also result in negatively charged Pd. This charge transfer was ascribed to the band filling modification and the orbital hybridization between substrate and metal atoms^40^. Specifically, for Pd deposited on magnesium termination, the surface Mg conduction band is shifted toward higher energy and is emptied, while the Pd *d* band is shifted in the opposite direction and becomes filled. Similar band filling modification and orbital hybridization might also apply to Pd–Bi_2_O_3_ system. This allows the Bi_2_O_3_-to-Pd intra-cluster electron transfer through direct Pd–Bi bonding across the interface, which leads to a greater filling of high-lying *d* bands in Pd clusters and largely circumvented over-hydrogenation problem. It is important to note that the charge transfer between Pd and Bi is localized in the Pd–Bi interface. Considering the relatively higher concentration of Bi_2_O_3_ comparing with Pd, the signal of electron-deficient Bi is likely overwhelmed by the signal of unaffected Bi_2_O_3_ and is therefore not observed by XPS and XANES.

The unique atomic and electronic structures of heterografted Pd cluster further result in its largely modulated gas adsorption behaviors compared with other supported Pd metals, which can be investigated by the in situ diffuse reflectance infrared Fourier transform spectroscopy using CO as the probe molecule. As shown in Fig. 2f, signals at 2089 and 2059 cm^−1^ for linear-bonded CO^43^ over Pd/TiO_2_ redshift to 2076 and 2055 cm^−1^ for those over Pd_1.0_/Bi_2_O_3_/TiO_2_, suggesting a strengthened π-back donation of metal *d*-electrons to π* orbitals of CO over Pd_1.0_/Bi_2_O_3_/TiO_2_ and a downshift of *d*-band center^44^. This result is well consistent with the XPS and XANES results. It is noteworthy that signals for bridge-bonded CO (1996 and 1948 cm^−1^) are observed over Pd/TiO_2_ but invisible over Pd_1.0_/Bi_2_O_3_/TiO_2_, likely arising from combined size and electronic effects. Specifically, the downshifted *d*-band center weakens the adsorption strength of CO on Pd^44^. More importantly, the small size and large structural distortion of Pd clusters disfavor the bridge adsorption mode of CO molecules due to the low average CN and broad distance distribution of Pd–Pd pairs.

### Catalytic performance in acetylene hydrogenation

With the unique hybrid cluster structure and Bi_2_O_3_-to-Pd intra-cluster electron transfer, Pd_1.0_/Bi_2_O_3_/TiO_2_ readily serves as a model catalyst to demonstrate the essential catalytic role of a nanometer metal/oxide interface. The catalytic properties were evaluated in selective hydrogenation of acetylene with excess ethylene, mimicking the front-end condition. In this condition, the thermodynamically favored over-hydrogenation of ethylene with the large excess hydrogen generally leads to an unsatisfied C_2_H_4_ selectivity at high C_2_H_2_ conversion, accompanied by a thermal runaway. To overcome this problem, a small amount of CO is usually added in the feed stream to reduce the reaction rate so as to improve the C_2_H_4_ selectivity^10^. At very low CO levels, high C_2_H_4_ selectivity and C_2_H_2_ conversion are difficult to achieve simultaneously. In this study, no CO is added in the feed stream. Both Pd/TiO_2_ and well-established Pd_1_Ag_3_/Al_2_O_3_ catalysts^30,45^ were evaluated for comparison with Pd_1.0_/Bi_2_O_3_/TiO_2_. The composition and synthesis procedure of the PdAg_3_/Al_2_O_3_ catalyst is the same as OleMax@251, a widely used industrial catalyst for acetylene hydrogenation^45^. The carbon balances are all >99%. Negligible oligomers were formed during the hydrogenation process, likely due to the short contact time and high concentration of hydrogen. According to the literatures, the large excess of hydrogen would change the adsorption modes of C_2_H_2_ and C_2_H_4_ from C-CH_2_ vinylidene and C-CH_3_ ethylidyne to weak π-bonded C_2_H_2_ and C_2_H_4_^46,47^. As a result, the possible reaction between vinylidene and acetylene to form C_4_ species as well as the hydrocarbon isomerization and decomposition are suppressed.

**Fig. 3:**
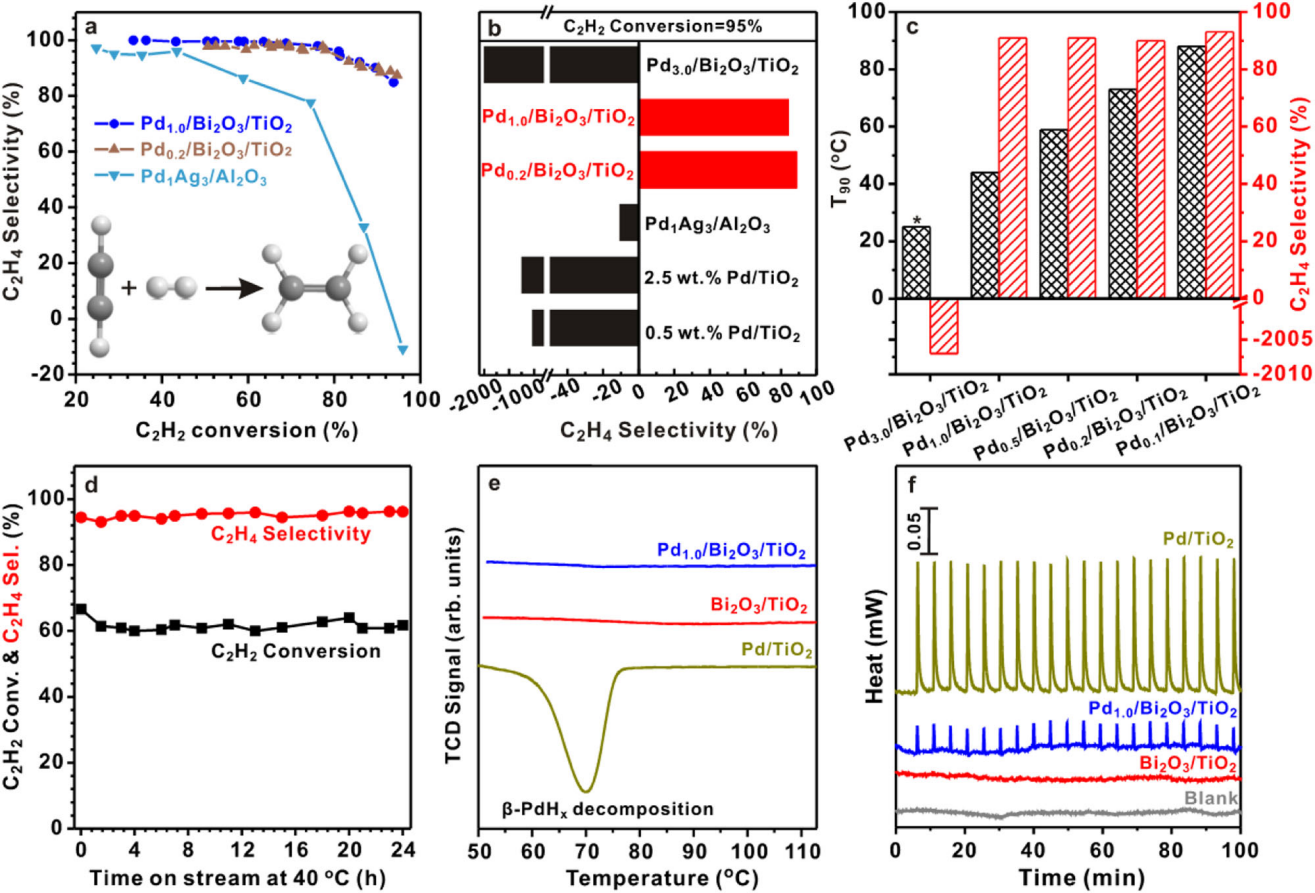
Catalytic performances of Pd_1.0_/Bi_2_O_3_/TiO_2_ in acetylene hydrogenation. **a** Selectivity as a function of acetylene conversion over Pd_1.0_/Bi_2_O_3_/TiO_2_, Pd_0.2_/Bi_2_O_3_/TiO_2_, and PdAg_3_/Al_2_O_3_. **b** The selectivity to C_2_H_4_ for 95% acetylene conversion over different catalysts. **c** Reaction temperature (*T*_90_) and C_2_H_4_ selectivity for 90% acetylene conversion. *For Pd_3.0_/Bi_2_O_3_/TiO_2_, hydrogen dissociation easily takes place at room temperature. The strong exothermic effect of unselective acetylene hydrogenation eventually leads to a runaway temperature up to 63.5 °C. **d** C_2_H_2_ conversion and C_2_H_4_ selectivity with time on stream over Pd_1.0_/Bi_2_O_3_/TiO_2_ at 40 °C. **e** H_2_-TPR profiles for Pd/TiO_2_, Bi_2_O_3_/TiO_2_, and Pd_1.0_/Bi_2_O_3_/TiO_2_. **f** Microcalorimetric studies of C_2_H_4_ pulse adsorption over Pd/TiO_2_ and Pd_1.0_/Bi_2_O_3_/TiO_2_. Source data are provided in a Source data file.

Figure 3a plots the C_2_H_4_ selectivity as a function of C_2_H_2_ conversion on various catalysts. Consistent with the literature results^30^, the C_2_H_4_ selectivity drops rapidly on well-established Pd_1_Ag_3_/Al_2_O_3_ catalyst once the C_2_H_2_ conversion exceeds 40%. On the contrary, Pd_1.0_/Bi_2_O_3_/TiO_2_ and Pd_0.2_/Bi_2_O_3_/TiO_2_ catalysts preserve very high selectivity toward C_2_H_4_ at much higher C_2_H_2_ conversions. Figure 3b compares the C_2_H_4_ selectivity at 95% C_2_H_2_ conversion. Interestingly, all catalysts except Pd_1.0_/Bi_2_O_3_/TiO_2_ and Pd_0.2_/Bi_2_O_3_/TiO_2_ exhibit negative selectivity toward C_2_H_4_ due to the over-hydrogenation of ethylene to ethane. Specifically, Pd_1.0_/Bi_2_O_3_/TiO_2_ exhibits much higher selectivity than 2.3 wt.% Pd/TiO_2_ regardless of the same Pd loading. In addition, we also synthesized Bi_*x*_/Pd/TiO_2_ (*x* = 0.5, 1, *x* is the molar ratio of Bi to Pd) by photo-depositing Bi onto 2.3 wt% Pd/TiO_2_. Interestingly, both Bi_0.5_/Pd/TiO_2_ and Bi_1.0_/Pd/TiO_2_ exhibit 100% conversion of C_2_H_2_ and negative selectivity toward ethylene (−123% for Bi_0.5_/Pd/TiO_2_ and −143% for Bi_0.5_/Pd/TiO_2_) at RT. These results suggest that a simple site blocking mechanism cannot explain the beneficial effect of Bi in this study. PdBi/TiO_2_ composed of PdBi IMC (Supplementary Fig. 9) shows 100% conversion of C_2_H_2_ and negative selectivity toward ethylene (−1319%) at RT. The strong exothermic effect of unselective acetylene hydrogenation eventually leads to a runaway temperature up to 58.5 °C. These results exclude PdBi IMC as the active site for Pd_1.0_/Bi_2_O_3_/TiO_2_ and further indicates the critical role of the nanometer Pd/Bi_2_O_3_ interface in the catalytic selectivity of Pd. It is important to highlight that 91% selectivity of C_2_H_4_ with 90% conversion of C_2_H_2_ is achieved by Pd_1.0_/Bi_2_O_3_/TiO_2_ at a temperature as low as 44 °C (Fig. 3c). Such an excellent low-temperature performance of acetylene hydrogenation has never been reported previously, suggesting the unique structure and catalytic properties of Pd_1.0_/Bi_2_O_3_/TiO_2_. Moreover, the C_2_H_2_ conversion and C_2_H_4_ selectivity remain almost constant over 24 h operating at 40 °C (Fig. 3d). XRD and XAFS also confirm that Pd/Bi_2_O_3_ hybrid cluster structure is still maintained (Fig. 2), demonstrating a good long-term stability of Pd_1.0_/Bi_2_O_3_/TiO_2_.

The variation of Pd-to-Bi ratio leads to an evolution of Pd/Bi_2_O_3_ hybrid clusters and major alteration of their catalytic performances in selective acetylene hydrogenation. Usually, the high Pd-to-Bi ratio results in the formation of Pd nanoparticles along with decreased Pd/Bi_2_O_3_ clusters, while low Pd-to-Bi ratio results in Pd single atoms. Here the reaction temperature (*T*_90_) and C_2_H_4_ selectivity at 90% conversion of acetylene are utilized to evaluate the catalytic performance of the samples. As shown in Fig. 3c, *T*_90_ increases along with the decrease of Pd-to-Bi ratio, implying that small Pd size is unfavorable to hydrogenation. Under an extreme condition when Pd is atomically dispersed (Pd-to-Bi ratio ≤0.1), a very high *T*_90_ (>90 °C) will be obtained, likely due to its poor ability of hydrogen activation^21^. In contrast, the moderate size of Pd in the hybrid clusters allows the efficient activation of hydrogen at much lower temperatures. All these catalysts exhibit quite high C_2_H_4_ selectivity. The Pd_3.0_/Bi_2_O_3_/TiO_2_ with an increased Pd-to-Bi ratio generates Pd nanoparticles and can even convert >90% of acetylene at RT. However, it suffers from the negative C_2_H_4_ selectivity at 90% conversion of acetylene. The above results experimentally confirm the trade-off between the conversion of acetylene and the selectivity of C_2_H_4_ upon the size effect of Pd, while an optimal catalytic performance is achieved over the Pd clusters stabilized by a nanometer metal–oxide interface.

The origin of such inherent trade-off in the catalytic performance of Pd lies in two aspects: the hydrogen activation and ethylene adsorption. It is generally accepted that the facile dissociative activation of hydrogen on Pd nanoparticles produces too much active H species, which migrate into the Pd lattice and generate β-hydride phase that leads to over-hydrogenation of ethylene^43^. Accordingly, a negative peak (55–75 °C, Fig. 3e) characteristic for the decomposition of β-hydride phase is observed from the temperature programmed reduction (TPR) profile of Pd/TiO_2_, which is, however, not observed from that of Pd_1.0_/Bi_2_O_3_/TiO_2_. These results are consistent with literatures that report high-coordinated Pd ensembles (the case of Pd/TiO_2_) as active sites for the formation of β-hydride^43^. As for Pd_1.0_/Bi_2_O_3_/TiO_2_, the Pd/Bi_2_O_3_ hybrid clusters feature a small Pd–Pd CN and nanometer Pd–Bi interface, which prevent the formation of β-hydride. The lack of β-hydride suppresses the over-hydrogenation of ethylene to ethane and therefore contribute to the high C_2_H_4_ selectivity^48^. It is important to highlight that the reduced size of Pd cluster does not compromise the hydrogen activation and thus the catalytic activity. As a result, a low *T*_90_ could be achieved on Pd_1.0_/Bi_2_O_3_/TiO_2_ (Fig. 3c).

In addition to the formation of β-hydride, the adsorption behavior of ethylene is also strongly influenced by the Pd structures, which leads to different microcalorimetric profiles^49^. Figure 3f shows the heat flow during the C_2_H_4_ pulse adsorption process as a function of time for Pd_1.0_/Bi_2_O_3_/TiO_2_, Pd/TiO_2_, and Bi_2_O_3_/TiO_2_ at 40 °C. Obvious heat flow signals are observed on Pd/TiO_2_ and Pd_1.0_/Bi_2_O_3_/TiO_2_ but are absent on Bi_2_O_3_/TiO_2_ and blank test, demonstrating that C_2_H_4_ is adsorbed on Pd instead of on Bi_2_O_3_. It is interesting to note that the amplitude of the heat flow signal of Pd_1.0_/Bi_2_O_3_/TiO_2_ is much smaller than that of Pd/TiO_2_.The calculated adsorption heat of Pd_1.0_/Bi_2_O_3_/TiO_2_ (~5.9 kJ mol^-1^) is significantly lower than that of Pd/TiO_2_ (~234.5 kJ mol^−1^), clearly indicating a much weaker ethylene adsorption on Pd_1.0_/Bi_2_O_3_/TiO_2_. These observations can be attributed to the unique geometric and electronic structure of hybrid cluster, similar to the results reported in the alloying of Pd^50,51^. The low Pd–Pd coordination and the Bi_2_O_3_-to-Pd intra-cluster electron transfer likely change the adsorption configuration of C_2_H_4_ from stable ethylidyne to weak π-bonded C_2_H_4_ and promote the desorption of ethylene as the desired product. To confirm this hypothesis, we further performed the temperature programmed desorption of ethylene by monitoring the mass signal of *m*/*e* = 27 (Supplementary Fig. 12). According to the literature, the peak at ~65 °C could be assigned to weak π-bonded ethylene species, which readily desorb without decomposition^52^. The peak centered at ~115 °C originates from di-σ-bonded ethylene, which undergoes decomposition followed by the recombination of surface hydrocarbon species and hydrogen to produce ethylene and ethane^52^. Compared with Pd/TiO_2_, Pd_1.0_/Bi_2_O_3_/TiO_2_ exhibits a much weaker peak at ~115 °C but a significantly larger peak at 65 °C. These results confirm that the adsorption configuration of C_2_H_4_ is changed from the strong σ-bonding for Pd/TiO_2_ to weak π-bonding for Pd_1.0_/Bi_2_O_3_/TiO_2_.

### Reaction mechanism investigated by DFT calculations

**Fig. 4:**
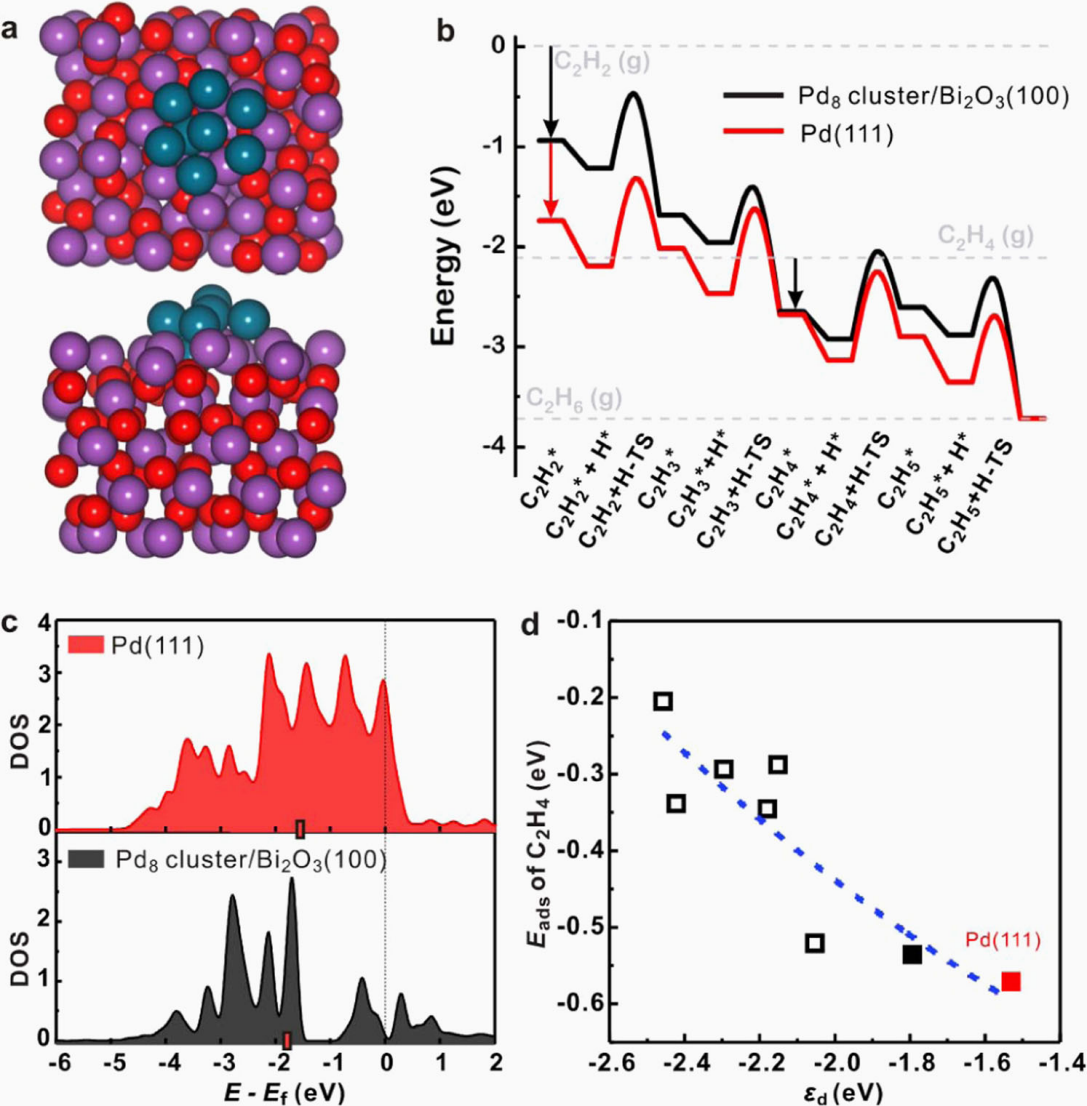
Reaction mechanism revealed by DFT calculations. **a** Optimized Pd cluster structure for DFT calculation (Pd: cyan, Bi: purple, O: red) and **b** energy profile of acetylene hydrogenation to ethane on Pd(111) and Pd_8_ cluster supported on Bi_2_O_3_(100). **c** DOS projected onto *d* electrons over Pd atom of Pd(111) and Pd_8_ cluster structures. A surface Pd atom of Pd(111), and the most active Pd atom of Pd_8_ cluster structure (on which C_2_H_4_ adsorbs most strongly) are chosen to plot the DOS. The position of *d*-band center (*ε*_d_) is highlighted with a red bar. **d** *E*_ads_ of C_2_H_4_ as a function of *ε*_d_ over different Pd atom on Pd cluster surface (black squares). The most stable adsorption configuration is shown as solid square, while the other less stable adsorption structures are denoted by hollow squares. A surface Pd atom of Pd(111) is also shown as red solid square for comparison. The blue fitted line is a guide for the eyes. It shows that a more negative *ε*_d_ corresponds to a more positive *E*_ads_ of C_2_H_4_. Source data are provided in a Source data file.

DFT calculations were performed to further provide insights into the molecular-level mechanisms of acetylene hydrogenation on experimental Pd_1.0_/Bi_2_O_3_/TiO_2_ catalyst. Model of Bi_2_O_3_-supported Pd_8_ cluster catalyst was built according to the experimental characterization results and the structure of which is shown in Fig. 4a (please see the details of model development in Supplementary Information). In this model, the size of Pd cluster on Bi_2_O_3_(100) is around 1.6 nm × 1.5 nm. In addition, this Pd cluster shows an average Pd–Pd CN of 4.0, which is close to the experimental values measured, i.e., 4.7 ± 0.5 (Supplementary Table 2). The Pd–Bi pair distribution function of the Pd_8_ cluster structure is shown in Supplementary Fig. 13. In this figure, the dominant peak appears at ~2.75 Å, which is smaller than that in the PdBi intermetallic model (~2.91 Å, Supplementary Table 3). In addition, Bader charge analysis suggests that Pd atoms in PdBi IMC (average charge of −0.36 e) model are more electron-rich than those in Pd-Bi_2_O_3_ hybrid clusters model (−0.21 e). These results are well consistent with Pd *K*-edge XANES and Pd 3*d* XPS shown in Supplementary Fig. 9, which therefore validate the reliability of the Pd_8_ cluster model. The possibility of hydride formation over the Pd cluster was studied, and it was found that hydrogen atoms prefer to adsorb at surface Pd sites after optimization (Supplementary Fig. 14), suggesting that formation of Pd hydride from this cluster is difficult, which again agrees well with the results shown in Fig. 3e.

It was reported that C-CH_2_ vinylidene and C-CH_3_ ethylidyne are energetic stable and important spectator species in acetylene hydrogenation^53–55^. However, in the front-end condition where H_2_ is in large excess, vinylidene and ethylidyne are readily hydrogenated and are insignificant at steady-state conditions^10^. Besides, the adsorption configurations of C_2_H_2_ and C_2_H_4_ also depend on the catalyst structure. For the Pd cluster structure studied in this work, it is found that the hydrogenation of CH≡CH to CHCH_2_ has the lowest activation energy (*E*_a_) of 0.74 eV compared with the dehydrogenation or hydrogen shift of CH≡CH (Supplementary Table 4). This strongly suggests that formation of CCH_2_ would be unfavorable on the Pd cluster structure. Similarly, the hydrogenation of CHCH_2_ to CH_2_CH_2_ has the lowest *E*_a_ among all reactions starting from CHCH_2_, again indicating that the spectator species CCH_3_ is hard to form on Pd cluster model (please see Supplementary Information for details). To this end, the effects of these species are not discussed here. The energy profile of acetylene hydrogenation to ethane over this Pd cluster is shown in Fig. 4b, and the corresponding optimized configurations of surface intermediates and transition states are shown in Supplementary Fig. 15. Meanwhile, the energy profile on Pd(111) representing Pd foil is also shown in Fig. 4b for comparison. The calculated barriers are generally consistent with the reported values (Supplementary Table 5), demonstrating that our calculated results are reliable. In addition, we further calculated the vibrational frequency based on the transition state structures on Pd(111). All the transition states were characterized to possess only one imaginary frequency along the bond formation of hydrogenation reactions. These results further demonstrate the reliability of the constrained minimization method that we used in this work. As can be seen from Fig. 4b, the adsorption of C_2_H_2_ over Pd(111) and Pd_8_ cluster is exothermic, and the transition state energies of C_2_H_2_ hydrogenation to C_2_H_4_ on both surfaces are below the energy of gaseous C_2_H_2_, suggesting that the hydrogenation processes should be facile. The semi-hydrogenation product C_2_H_4_ would either desorb from the surface or undergo further hydrogenation to ethane (C_2_H_6_). In Fig. 4b, the transition state energy of C_2_H_4_ hydrogenation on Pd_8_ cluster is above the gaseous C_2_H_4_ energy, suggesting that desorption of C_2_H_4_ from the Pd_8_ cluster may be favored compared with its further hydrogenation to C_2_H_5_. Herein the difference between the barriers for further hydrogenation and desorption of ethylene can be used to estimate the possibility of selective C_2_H_4_ formation^17,56–60^. Within this framework, a more positive value of *E*_a,hydro_ − |*E*_ads,C2H4_|, where *E*_a,hydro_ is the effective hydrogenation barrier of C_2_H_4_ to C_2_H_6_ and |E_ads,C2H4_| is the absolute value of C_2_H_4_ adsorption energy, corresponds to higher C_2_H_4_ selectivity. The calculated values of *E*_a,hydro_ − |E_ads,C2H4_| over Pd_8_ cluster and Pd(111) are 0.53 and 0.31 eV, respectively, demonstrating higher C_2_H_4_ selectivity over the Pd_8_ cluster than Pd(111), which is consistent with the experimental results.

We further carried out electronic structure analysis to understand the weaker adsorption of reaction intermediates over the Pd cluster than Pd(111). In Fig. 4c, we plotted the density of states projected onto *d*-electrons of the surface Pd atom where C_2_H_4_ adsorbs over Pd(111) and Pd cluster, and the *d*-band center (*ε*_d_) were calculated to be −1.53 and −1.79 eV, respectively. According to the *d*-band center theory^61^, the more negative *ε*_d_ indicates weaker binding to adsorbates, which is in agreement with the trend of adsorption energies calculated for the reaction intermediates over Pd(111) and Pd cluster. In addition, the *ε*_d_ of each Pd atom on Pd cluster was found to be correlated with *E*_ads_ of C_2_H_4_ as shown in Fig. 4d, and a more negative *ε*_d_ generally corresponds to weaker adsorption of C_2_H_4_. The most stable adsorption of ethylene, corresponding to the black solid square in Fig. 4d and *C_2_H_4_ in Fig. 4b, gives rise to the *E*_ads_ of −0.54 eV, which is slightly weaker than that over Pd(111), i.e., −0.57 eV. However, one can also see that C_2_H_4_ adsorbs much weaker at other Pd sites over the Pd_8_ cluster (shown as black hollow squares), therefore Pd_8_ cluster shows weaker adsorption on average compared with those sites over Pd(111) where all the surface sites are identical. C_2_H_4_ would be more likely to desorb from the Pd cluster, resulting in the high C_2_H_4_ selectivity observed.

Our findings demonstrate the essential catalytic roles of a nanometer metal/oxide interface in selective hydrogenation of acetylene. Pd–Bi_2_O_3_ hybrid clusters feature a small Pd–Pd CN and intra-cluster electron transfer, which enables a weak C_2_H_4_ adsorption without compromising the H_2_ activation activity. The superior low-temperature catalytic performance of Pd–Bi_2_O_3_ nanocluster ensembles over Pd single atom and nanoparticles might open new opportunities for fundamental research of hybrid nanoclusters. Besides, the demonstrated stepwise photochemical strategy also provides a new path for fabricating hybrid nanoclusters and nanometer metal/oxide interface.

## Methods

### Synthesis of Pd_*x*_/Bi_2_O_3_/TiO_2_

Pd_*x*_/Bi_2_O_3_/TiO_2_ (*x* is the nominal molar ratio of Pd to Bi) catalysts were prepared by a two-step photo-deposition method using a high-pressure Xe lamp (300 W) as the light source. Typically, 100 mg of TiO_2_ and 11.6 mg of Bi(NO_3_)_3_·5H_2_O (Sinopharm chemicals, 99%) were dispersed in 4 mL of ethylene glycol in a Pyrex glass reactor. Prior to ultraviolet irradiation for 1 h, the suspension was bubbled with Ar for 30 min to eliminate dissolved O_2_. Subsequently, 8 mL of PdCl_2_ aqueous solution (the concentration is determined by *x*) was added into the suspension. After irradiation in Ar for another 1 h, the precipitates were collected by centrifugation, washed twice by water and ethanol, and then dried in an oven at 40 °C. When the sample was exposed in the air, Bi was oxidized to Bi_2_O_3_ spontaneously. The catalyst was activated by H_2_ at 100 °C for 1 h and then cooled to RT in N_2_ prior to the catalytic reaction and characterizations. The synthetic procedures of Bi_2_O_3_/TiO_2_, Pd/TiO_2_, Pd_1.0_/Bi_2_O_3_/TiO_2_-ox, PdBi/TiO_2_, and Pd/Bi_2_O_3_ are presented in Supplementary Information.

### Characterization

Powder XRD patterns were recorded on a Rigaku Ultimate IV diffractometer using Cu Kα radiation operated at 40 mA and 40 kV (scan rate: 5^o^ min^−1^). XPS measurements were performed in a VG Scientific ESCALAB Mark II spectrometer. All BEs were referenced to the C 1s peak at 284.8 eV of the surface adventitious carbon to correct the shift caused by charge effect. In situ near ambient pressure XPS (NAP-XPS) was conducted using a Specs NAP-XPS system with a PHOIBOS NAP hemispherical energy analyzer in Vacuum Interconnected Nanotech Workstation, Suzhou Institute of Nano-Tech and Nano-Bionics. The NAP operation was conducted under 1 mbar H_2_ atmosphere. The actual loading of Pd and Bi were analyzed by ICP-AES using a Profile Spec ICP-AES spectrometer (Leeman, USA). Structural characterizations and chemical composition distribution were determined by a probe Cs-corrected electron microscope (FEI Titan) equipped with a Super-X EDS. Microcalorimetric measurements of C_2_H_4_ adsorption were carried out on a home-designed adsorption microcalorimetry system consisting of a chemisorption apparatus (Micromeritics Autochem II 2920) and a microcalorimeter (Setaram Sensys EVO 600).

The XAFS spectra at Pd *K*-edge and Bi L3-edge of the samples were measured at beamline 14W of Shanghai Synchrotron Radiation Facility in China^62^. The output beam was filtered by Si(311) monochromator. Pd foil was used to calibrate the energy.

In situ Fourier transform infrared (FT-IR) adsorption spectroscopy of CO experiments were recorded on a Nicolet iS50 instrument. Prior to CO chemisorption, the catalysts were activated by H_2_ at 100 °C for 1 h and then cooled down to RT in Ar. The FT-IR spectrum of Ar at RT was taken as the background spectrum and subtracted automatically from subsequent spectra. Then the catalysts were exposed to a CO flow for 10 min and degassed by Ar for 10 min to desorb the physical adsorbed CO, and IR spectra were recorded.

TPR measurements were conducted on Micromeritics ChemiSorb 2920, equipped with a thermal conductivity detector. Prior to TPR measurements, 100 mg of the catalyst was activated by H_2_ at 100 °C for 1 h and then cooled down to RT in Ar. Afterwards, the catalyst was subjected to 10 vol% H_2_/Ar at a flow rate of 30 mL min^−1^ and heated to 150 °C at 10 °C min^−1^.

### Catalytic tests

Selective hydrogenation of acetylene in excess ethylene was carried out in a fixed bed vertical quartz reactor, with a space velocity of 120,000 mL h^–1^ g^–1^. The reaction gas consisting of 1.0 vol% C_2_H_2_, 20.0 vol% C_2_H_4_, 20.0 vol.% H_2_, and 59 vol.% N_2_ was fed at a flow rate of 60 mL min^−1^, simulating the front-end hydrogenation conditions. Typically, 30 mg of the catalyst (diluted by 400 mg of quartz sand) was activated by H_2_ (20 mL min^–1^) at 100 °C for 1 h and then cooled to RT in N_2_ prior to the catalytic reaction. The gas components from the microreactor outlet were analyzed by online gas chromatography (GC; Shimadzu GC-2010) equipped with a flame ionization detector. It is important to note that the pretreatment in H_2_ is important as the long-term exposure of Pd_1.0_/Bi_2_O_3_/TiO_2_ in air might oxidize Pd and demolish Pd–Bi bonding. Control experiment also suggests that untreated Pd_1.0_/Bi_2_O_3_/TiO_2_ has poor selectivity toward ethylene. To this end, all catalysts were pretreated in H_2_ before catalytic measurements.

C_2_H_4_ and C_2_H_6_ were the only products detected by GC. Negligible oligomers were formed during the hydrogenation process, likely due to the short contact time and high concentration of hydrogen^10^. C_2_H_2_ conversion and C_2_H_4_ selectivity were calculated as:

1 $${{{{{{\rm{C}}}}}}}_{2}{{{{{{\rm{H}}}}}}}_{2}\;{{{{{\rm{conversion}}}}}}=\frac{[{{{{{{\rm{C}}}}}}}_{2}{{{{{{\rm{H}}}}}}}_{2}]_{{{{{{\rm{inlet}}}}}}}-[{{{{{{\rm{C}}}}}}}_{2}{{{{{{\rm{H}}}}}}}_{2}]_{{{{{{\rm{outlet}}}}}}}}{[{{{{{{\rm{C}}}}}}}_{2}{{{{{{\rm{H}}}}}}}_{2}]_{{{{{{\rm{inlet}}}}}}}}\times 100 \%$$

2 $${{{{{{\rm{C}}}}}}}_{2}{{{{{{\rm{H}}}}}}}_{4}\;{{{{{\rm{selectivity}}}}}}=\frac{[{{{{{{\rm{C}}}}}}}_{2}{{{{{{\rm{H}}}}}}}_{2}]_{{{{{{\rm{inlet}}}}}}}-[{{{{{{\rm{C}}}}}}}_{2}{{{{{{\rm{H}}}}}}}_{2}]_{{{{{{\rm{outlet}}}}}}}-[{{{{{{\rm{C}}}}}}}_{2}{{{{{{\rm{H}}}}}}}_{6}]_{{{{{{\rm{outlet}}}}}}}}{[{{{{{{\rm{C}}}}}}}_{2}{{{{{{\rm{H}}}}}}}_{2}]_{{{{{{\rm{inlet}}}}}}}-[{{{{{{\rm{C}}}}}}}_{2}{{{{{{\rm{H}}}}}}}_{2}]_{{{{{{\rm{outlet}}}}}}}}\times 100 \%$$

### Computational details

In this work, Vienna Ab initio Simulation Package^63–66^ was used to conduct density functional calculations within the generalized gradient approximation of RPBE functional developed by the Nørskov group^67^. Ionic cores and electrons were described by the projector augmented wave method^68,69^. The energy cutoff was set to be 500 eV and the force threshold was 0.05 eV Å^−1^. We used constrained minimization method^59,70–72^ to locate the transition state structures. The adsorption energies of C_2_H_*x*_ (*x* = 2, 4, and 6) were calculated as: *E*_ads_ = *E*_C2H*x*+slab_ − (*E*_C2H*x*_ + *E*_slab_), where *E*_C2H*x*+slab_ is the energy of the system after adsorption of C_2_H_*x*_ species, *E*_C2H*x*_ is the energy of the gas-phase C_2_H_*x*_ adsorbate, and *E*_slab_ is the energy of the slab.

The optimized lattice parameters of Bi_2_O_3_ were *a* = 5.981 Å, *b* = 8.340 Å, and *c* = 7.591 Å, which are close to experimental values (monoclinic, *P*2_1_/c(14), *a* = 5.849 Å, *b* = 8.166 Å, and *c* = 7.510 Å). A Bi_2_O_3_(100) slab was built with a 2 × 2 supercell with nine atomic layers, and the Pd cluster structure was built by adding 8 Pd atoms onto the Bi_2_O_3_(100) surface, followed by structure optimization. To build a valid catalyst model of Pd cluster, we followed these rules: (i) Pd would disperse on the Bi_2_O_3_ surface with a size <2 nm; (ii) the structure should give rise to similar CNs listed in Supplementary Table 2; (iii) the model should be stable and would not deform under reaction condition, which has been widely discussed. During the optimizations involving Pd cluster structure, the bottom two layers of atoms of Bi_2_O_3_(100) component were fixed to simulate bulk structure of Bi_2_O_3_, while the other atoms were fully relaxed. The vacuum layer was set higher than 12 Å to avoid spurious interaction between adjacent slabs. The *k*-point grid used in the surface Brillouin zone was 1 × 1 × 1 for all the calculations. More details about the structure development are provided in Supplementary Information (Supplementary Figs. 16 and 17).

## Supplementary information

Supplementary Information

Peer Review File

## Data Availability

All the data supporting the findings of this study are available within the article and its Supplementary Information files or from the corresponding authors upon reasonable request. Source data are provided with this paper.

## Source data

Source Data
