# Supplementary material for: Grafting nanometer metal/oxide interface towards enhanced low-temperature acetylene semi-hydrogenation
Source: Nat Commun. 2021 Oct 1;12:5770. doi: 10.1038/s41467-021-25984-8 (PMC8486880; doi:10.1038/s41467-021-25984-8)
Supplement: Supplementary file 1 — Supplementary Information [file 41467_2021_25984_MOESM1_ESM.pdf]

Supplementary Information

Grafting nanometer metal/oxide interface towards enhanced low-temperature acetylene semi-hydrogenation

Shihui Zou[1†*], Baohui Lou[1†], Kunran Yang[2†], Wentao Yuan[3], Chongzhi Zhu[4], Yihan Zhu[4*], Yonghua Du[5,6*], Linfang Lu[1], Juanjuan Liu[7], Weixin Huang[8], Bo Yang[2*], Zhongmiao Gong[9], Yi Cui[9], Yong Wang[3], Lu Ma[6], Jingyuan Ma[10], Zheng Jiang[10], Liping Xiao[1], Jie Fan[1*]

[1]Key Lab of Applied Chemistry of Zhejiang Province, Department of Chemistry, Zhejiang University, Hangzhou 310036, China

[2]School of Physical Science and Technology, ShanghaiTech University, Shanghai 201210, China

[3]School of Materials Science and Engineering, Zhejiang University, Hangzhou 310027, China

[4]Center for Electron Microscopy, State Key Laboratory Breeding Base of Green Chemistry Synthesis Technology and College of Chemical Engineering, Zhejiang University of Technology, Hangzhou 310014, China

[5]Institute of Chemical and Engineering Sciences, A*STAR, Singapore 627833, Singapore

[6]National Synchrotron Light Source II, Brookhaven National Laboratory Upton NY, 11973 USA

[7]College of Materials & Environmental Engineering, Hangzhou Dianzi University, Hangzhou 310036, China

[8]Department of Chemical Physics, University of Science and Technology of China, Jinzhai Road 96, Hefei 230026, China

[9]Vacuum Interconnected Nanotech Workstation, Suzhou Institute of Nano-Tech and Nano-Bionics, Chinese Academy of Sciences, Suzhou 215123, China

[10]Shanghai Synchrotron Radiation Facility, Shanghai Institute of Applied Physics Chinese Academy of Sciences, Shanghai 201800, China

[†] These authors contributed equally to this work.

*Corresponding author. E-mail: xueshan199@163.com (S.Z.); jfan@zju.edu.cn (J.F.); yangbo1@shanghaitech.edu.cn (B.Y.); yihanzhu@zjut.edu.cn (Y.Z.); ydu@bnl.gov (Y.D.)

Supplementary Methods

Synthesis of Bi_2O_3/TiO_2. Bi_2O_3/TiO_2 was prepared by a photo-deposition method using a high-pressure Xe lamp (300 W) as the light source. Typically, 100 mg of TiO_2 and 11.6 mg of $Bi(NO_3)_3 \cdot 5H_2O$ (Sinopharm Chemicals, 99%) were dispersed in 4 mL of ethylene glycol in a Pyrex glass reactor. Prior to UV-irradiation for 1 h, the suspension was bubbled with Ar for 30 min to eliminate dissolved O_2. The precipitates were collected by centrifugation, washed twice by water and ethanol, and then dried in an oven at 40 °C. When the sample was exposed in the air, Bi was oxidized to Bi_2O_3 spontaneously.

Synthesis of Pd/TiO_2. Pd/TiO_2 was prepared by a photo-deposition method using a high-pressure Xe lamp (300 W) as the light source. Typically, 100 mg of TiO_2 was dispersed in 4 mL of ethylene glycol in a Pyrex glass reactor. Then, 8 mL of $PdCl_2$ aqueous solution (the concentration is determined by mass loading) was added into the suspension. The suspension was bubbled with Ar for 30 min to eliminate dissolved O_2. After irradiation for 1 h, the precipitates were collected by centrifugation, washed twice by water and ethanol, and then dried in an oven at 40 °C.

Synthesis of $Pd_{1.0}/Bi_2O_3/TiO_2$-ox. $Pd_{1.0}/Bi_2O_3/TiO_2$-ox was prepared by mildly oxidizing $Pd_{1.0}/Bi_2O_3/TiO_2$ in air at 150 °C for 1 h.

Synthesis of $PdBi/TiO_2$. $PdBi/TiO_2$ was prepared by a two-step deposition-reduction method using $NaBH_4$ as the reducing agent. Typically, 100 mg of TiO_2 and 11.6 mg of $Bi(NO_3)_3 \cdot 5H_2O$ were dispersed in 8 mL of ethylene glycol aqueous solution (50 vol.%). The suspension was stirred for 90 min before the dropwise addition of 2 mL of $NaBH_4$ aqueous solution (5 M). After stirred for another 30 min, 4 mL of acetone dissolving 5.4 mg of $Pd(OAc)_2$ was added to above suspension dropwise. The mixture was stirred for 1 h to allow a complete reduction of Pd. The precipitates were collected by centrifugation, washed twice by water and ethanol, and then dried in a vacuum oven at 40 °C. The catalysts were treated in H_2/Ar at 350 °C for 2 h before used for characterizations and catalytic tests. XRD patterns (Supplementary Fig. 9a) of $PdBi/TiO_2$ exhibit characteristic peaks at 39.9 and 42.8°, corresponding to (102) and

(110) of hexagonal PdBi intermetallic (sobolevskite, $P6_3$/mmc(194), a = b = 4.22 Å, c = 5.709 Å, α = β = 90°, and γ=120 °).

Synthesis of Pd/Bi_2O_3. Pd/Bi_2O_3 was prepared by a deposition-reduction method using $NaBH_4$ as the reducing agent. Typically, 100 mg of Bi_2O_3 was dispersed in 8 mL of water. Subsequently, 4 mL of acetone dissolving 5.4 mg of $Pd(OAc)_2$ was added to above suspension. The suspension was stirred for 10 min before the dropwise addition of 2 mL of $NaBH_4$ aqueous solution (5 M). The mixture was stirred for 1 h to allow a complete reduction of Pd. The precipitates were collected by centrifugation, washed twice by water and ethanol, and then dried in a vacuum oven at 40 °C. The catalyst was activated by H_2 at 100 °C for 1 h and then cooled to room temperature in N_2 prior to the catalytic reaction and characterizations.

Synthesis of $Bi_x/Pd/TiO_2$. $Bi_x/Pd/TiO_2$ (*x* is the nominal molar ratio of Bi-to-Pd, *x* = 0.5 or 1) catalysts were prepared by a two-step photo-deposition method using a high-pressure Xe lamp (300 W) as the light source. Typically, 100 mg of TiO_2 was dispersed in 4 mL of ethylene glycol in a Pyrex glass reactor. Then, 4 mL of $Pd(OAc)_2$ (5.4 mg) aqueous solution was added into the suspension. Prior to UV-irradiation for 1 h, the suspension was bubbled with Ar for 30 min to eliminate dissolved O_2. Subsequently, 4 mL of ethylene glycol dissolving certain amount of $Bi(NO_3)_3 \cdot 5H_2O$ (the concentration is determined by *x*) was added into the suspension. After irradiation in Ar for another 1 h, the precipitates were collected by centrifugation, washed twice by water and ethanol, and then dried in an oven at 40 °C. When the sample was exposed in the air, Bi was oxidized to Bi_2O_3 spontaneously. The catalyst was activated by H_2 at 100 °C for 1 h and then cooled to room temperature in N_2 prior to the catalytic reaction and characterizations.

ADF-STEM simulations. ADF-STEM simulations were carried out using the Dr. probe package. The parameters of simulations were considered based on HRSTEM experiments under 300 kV in the work. Specifically, convergence angle of 30 mrad, annular detector range of 70 – 150 mrad, third order spherical aberration (C3) of 1 μm and fifth order spherical aberration (C5) of 300 μm.

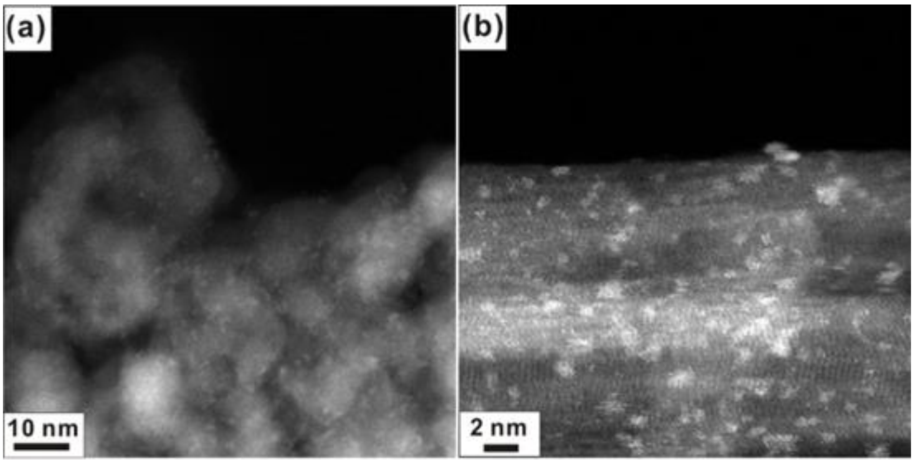

Supplementary Fig. 1 | STEM imaging of $Pd_{1.0}/Bi_2O_3/TiO_2$. (a, b) Low-magnification STEM images of $Pd_{1.0}/Bi_2O_3/TiO_2$.

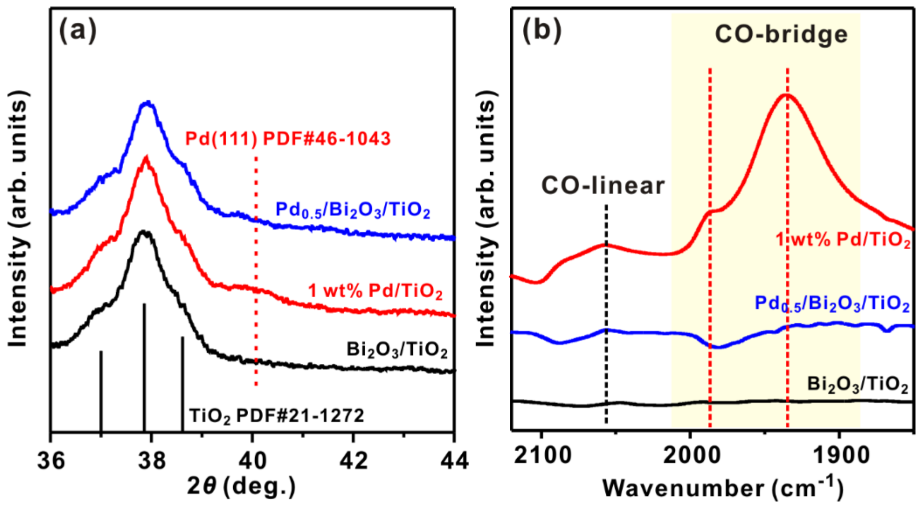

Supplementary Fig. 2 | Characterizations of $Pd_{0.5}/Bi_2O_3/TiO_2$. (a) XRD patterns; (b) CO-adsorbed FT-IR spectra. $Pd_{0.5}/Bi_2O_3/TiO_2$ exhibits no reflections associated with Pd, suggesting high dispersion of Pd species assisted by the pre-deposited Bi_2O_3 clusters in $Pd_{0.5}/Bi_2O_3/TiO_2$. The absence of bridge-bonded CO further confirms this result. Source data are provided in a Source Data file.

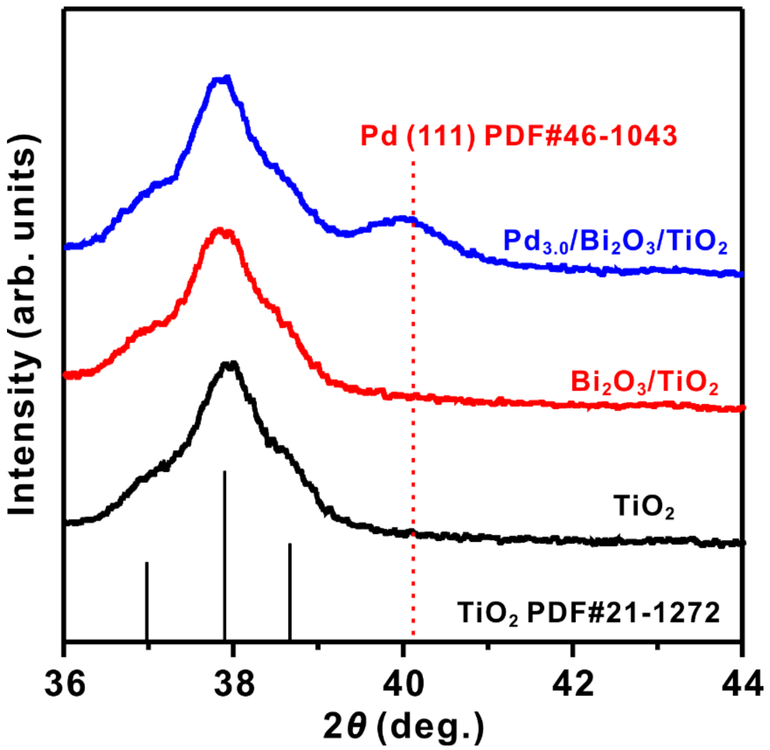

Supplementary Fig. 3 | XRD patterns of $Pd_{3.0}/Bi_2O_3/TiO_2$. A characteristic Pd(111) diffraction peak was observed at 40.1°, indicating the presence of Pd nanoparticles in $Pd_{3.0}/Bi_2O_3/TiO_2$. According to Scherrer equation, the average size of PdNPs in $Pd_{3.0}/Bi_2O_3/TiO_2$ is 6.7 nm. Source data are provided in a Source Data file.

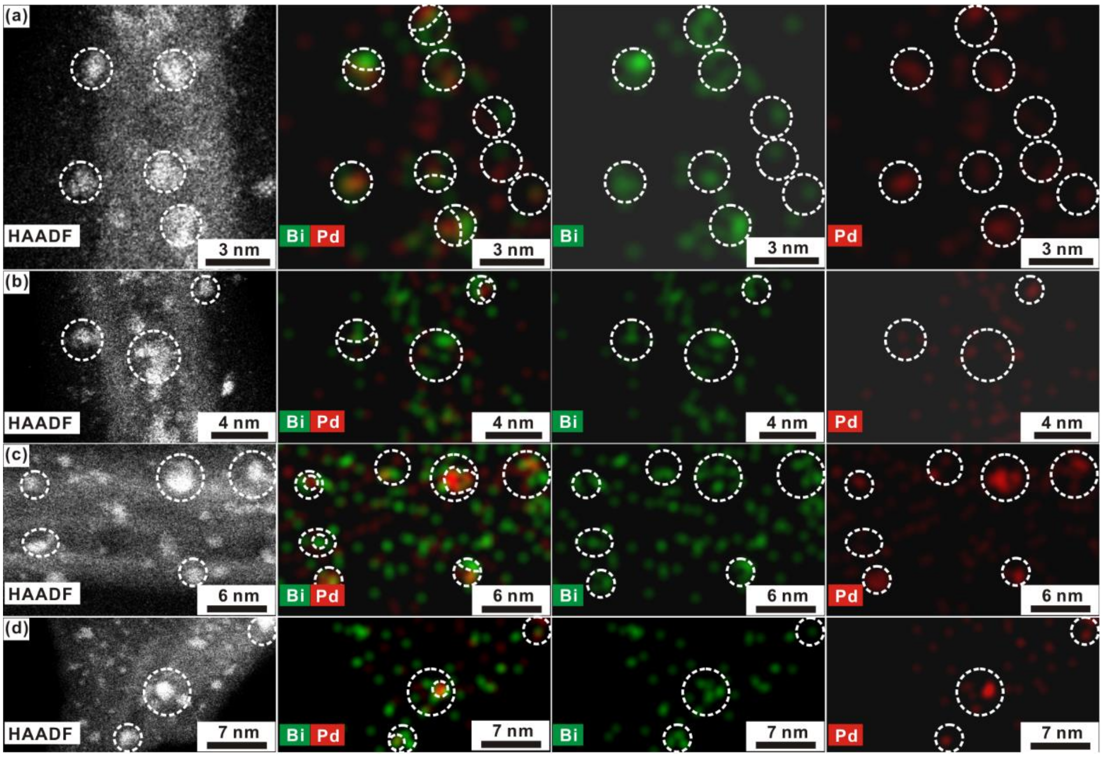

Supplementary Fig. 4 | Elemental mapping of $Pd_{1.0}/Bi_2O_3/TiO_2$. a, b, c, d represent four different regions.

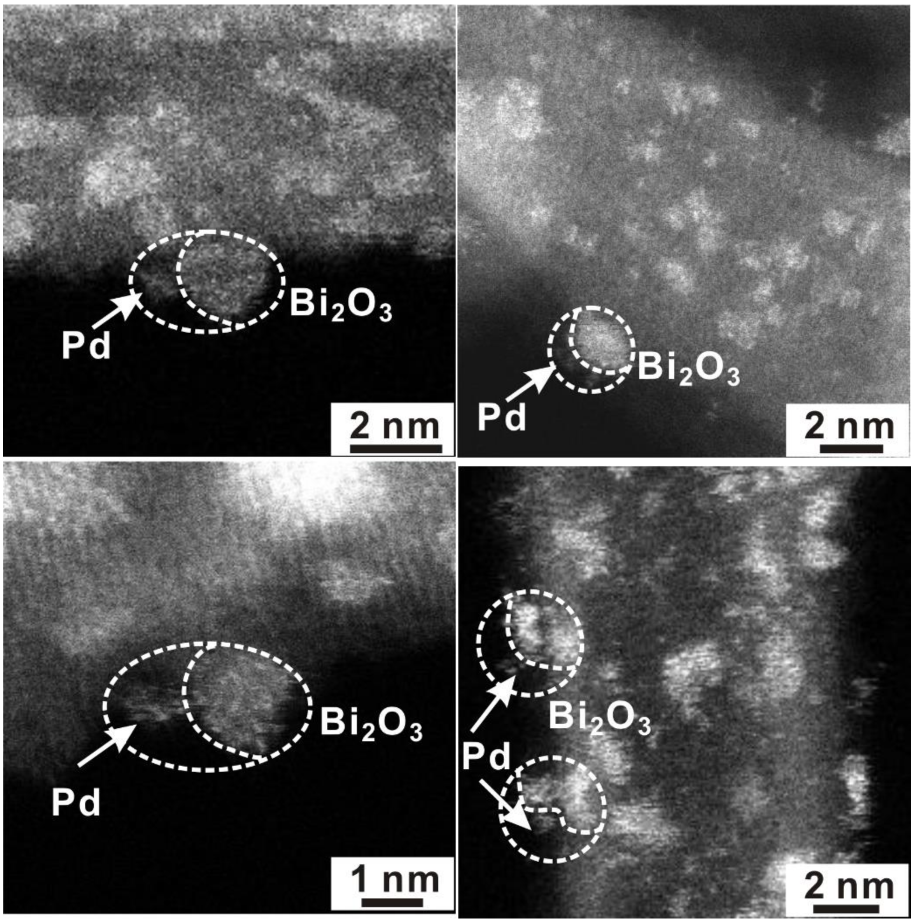

Supplementary Fig. 5 | HAADF-STEM images of $Pd_{1.0}/Bi_2O_3/TiO_2$. High-resolution STEM images of four different regions were presented.

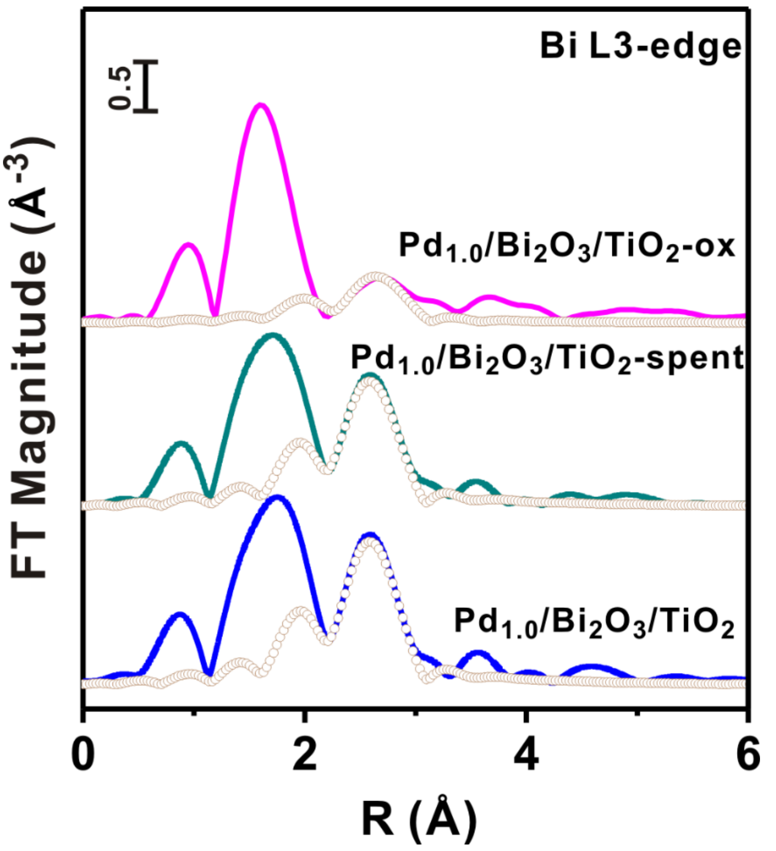

Supplementary Fig. 6 | Fourier transform spectra and fitting curves (without phase correction) of Bi-Pd shell in Bi L3 edge. Source data are provided in a Source Data file.

Supplementary Table 1. Structural Parameters of $Pd_{1.0}/Bi_2O_3/TiO_2$, $Pd_{1.0}/Bi_2O_3/TiO_2$-spent and $Pd_{1.0}/Bi_2O_3/TiO_2$-ox Extracted from the EXAFS Fitting of Bi L3 edge.

**Samples**	**Scattering pair**	**CN*a***	**R*b* (Å)**	**$\sigma^2 \times 10^2$(Å^2) *c***
$Pd_{1.0}/Bi_2O_3/TiO_2$	Bi-Pd	2.9±0.3	2.79±0.03	1.36±0.17
$Pd_{1.0}/Bi_2O_3/TiO_2$-spent	Bi-Pd	2.8±0.3	2.79±0.03	1.36±0.17
$Pd_{1.0}/Bi_2O_3/TiO_2$-ox	Bi-Pd	1.1±0.2	2.79±0.03	1.45±0. 20

[a]CN is the coordination number; [b]R is interatomic distance; [c]σ^2 is Debye-Waller factor (a measure of thermal and static disorder in absorber-scatter distances).

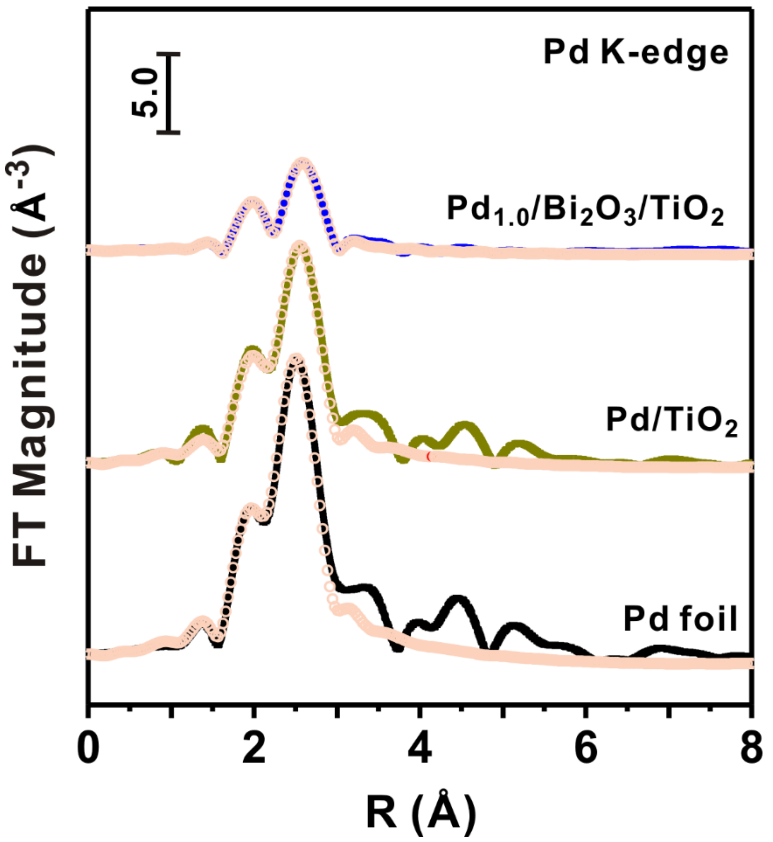

Supplementary Fig. 7 | Fourier transform spectra and fitting curves (without phase correction) of Pd foil, Pd/TiO_2 and $Pd_{1.0}/Bi_2O_3/TiO_2$ in Pd K edge. Source data are provided in a Source Data file.

Supplementary Table 2. Structural Parameters of Pd Foil, Pd/TiO_2 and $Pd_{1.0}/Bi_2O_3/TiO_2$ Extracted from the EXAFS Fitting of Pd K edge.

**Samples**	**Scattering pair**	**CN**[a]	**R**[b] **(Å)**	$\sigma^2 \times 10^2$**(Å²)**[c]
Pd foil	Pd-Pd	12[d]	2.74±0.01	0.55±0.06
Pd/TiO_2	Pd-Pd	10.0±1.0	2.74±0.01	0. 69±0.12
$Pd_{1.0}/Bi_2O_3/TiO_2$	Pd-Bi	4.6±0.5	2.79±0.02	1.77±0.18
	Pd-Pd	4.7±0.5	2.79±0.02	0.81±0.08

[a]CN is the coordination number; [b]R is interatomic distance; [c]σ^2 is Debye-Waller factor (a measure of thermal and static disorder in absorber-scatter distances); [d]This CN was fixed in the fitting.

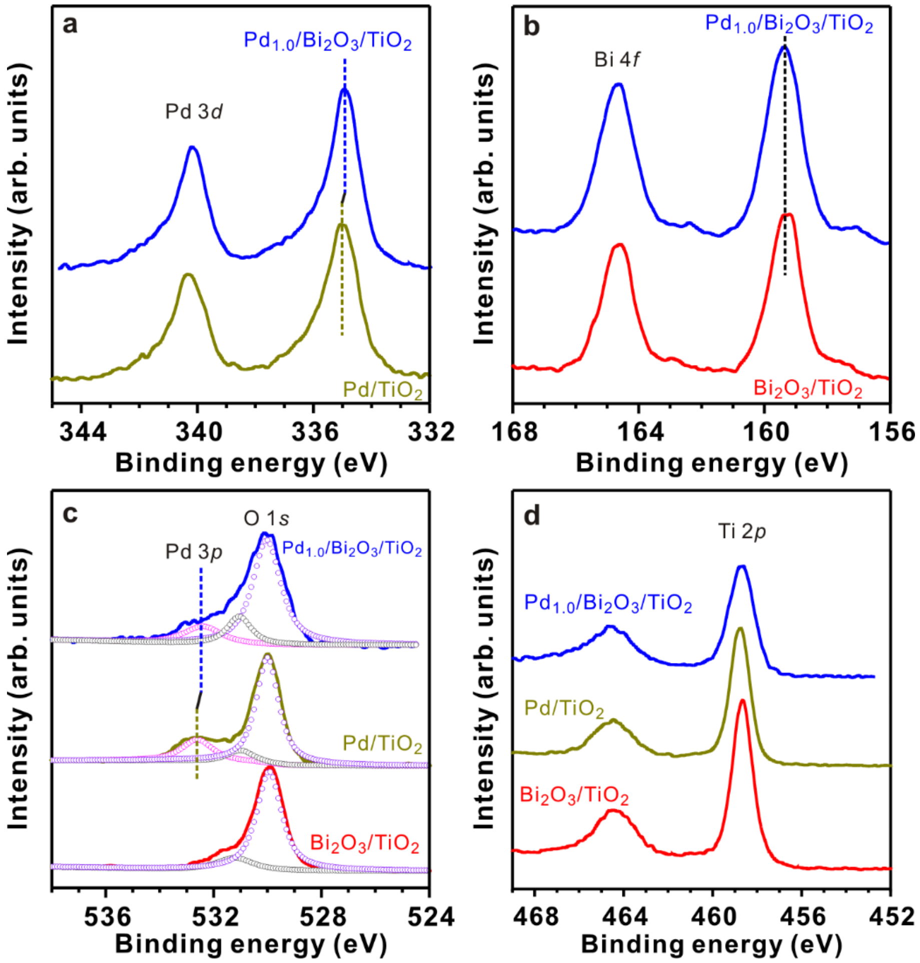

Supplementary Fig. 8 | In situ X-ray photoelectron spectra of $Pd_{1.0}/Bi_2O_3/TiO_2$, Bi_2O_3/TiO_2 and Pd/TiO_2 collected at 100 °C under H_2 atmosphere. (a) Pd 3*d*; (b) Bi 4*f*; (c) O 1*s*; (d) Ti 2*p*. Source data are provided in a Source Data file.

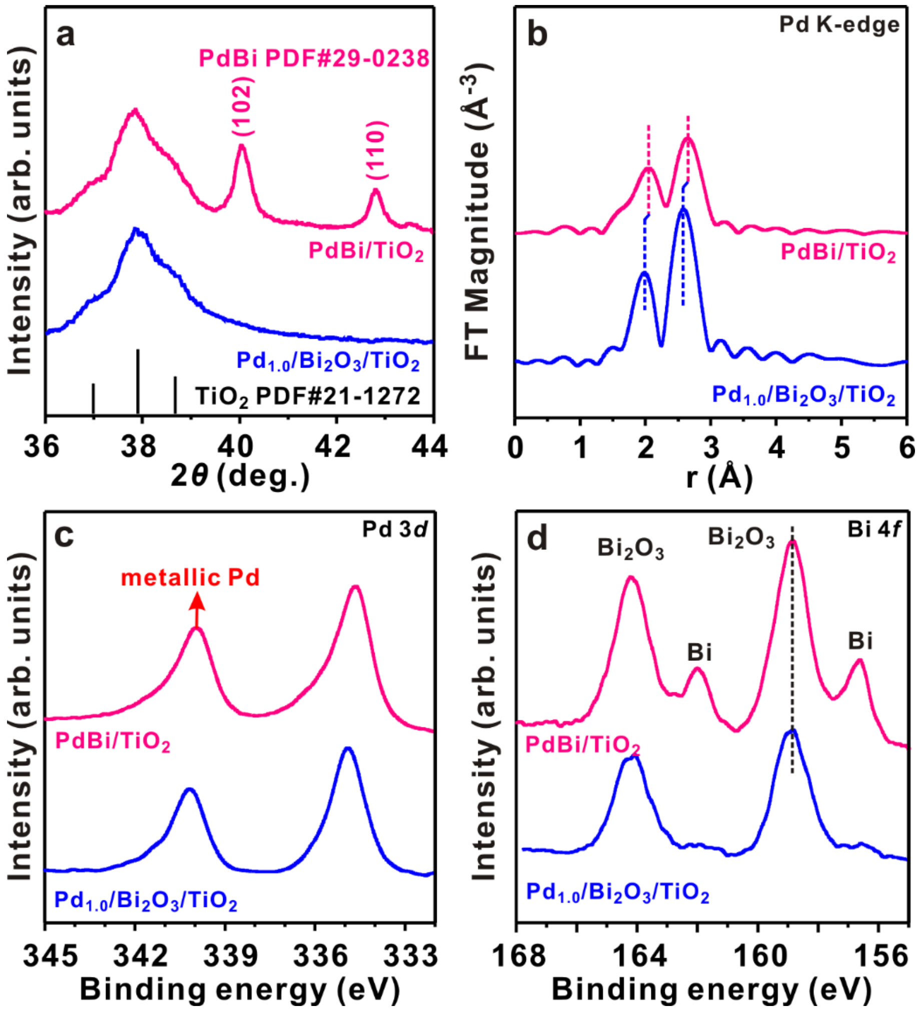

Supplementary Fig. 9 | Characterizations of PdBi/TiO_2. (a) XRD patterns; (b) Fourier transform spectra of Pd K-edge EXAFS; (c) Pd 3*d* XPS spectra; and (d) Bi 4*f* XPS spectra of $Pd_{1.0}/Bi_2O_3/TiO_2$ and PdBi/TiO_2. Source data are provided in a Source Data file.

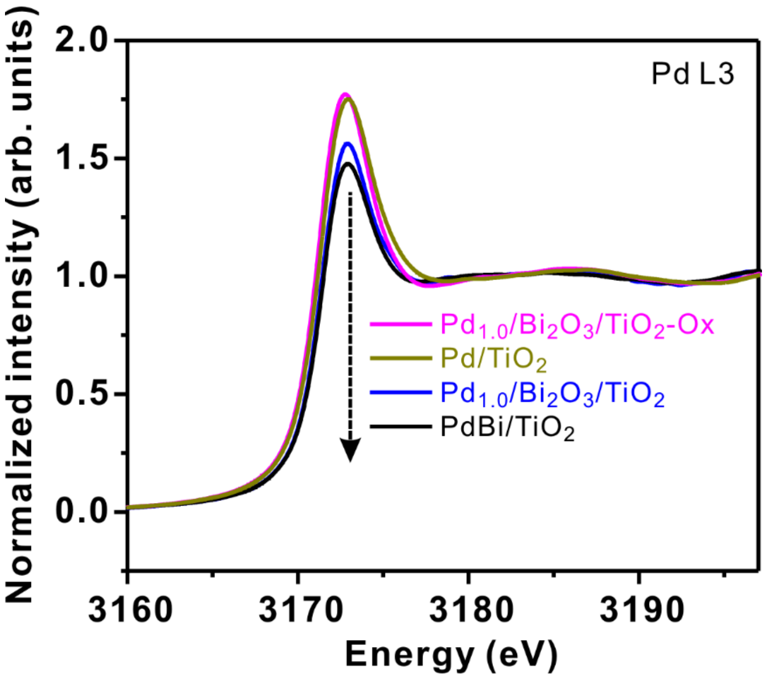

Supplementary Fig. 10 | Pd L3-edge XANES spectra for Pd/TiO_2, PdBi/TiO_2, $Pd_{1.0}$/Bi_2O_3/TiO_2 and $Pd_{1.0}$/Bi_2O_3/TiO_2-ox. Pd L3 near-edge absorption peak at ~3173 eV indicates the excitation of 2*p*3/2 electrons to the vacant 4*d*-states. A stronger absorption peak is expected for catalysts containing Pd atoms with more unfilled 4*d*-states. Source data are provided in a Source Data file.

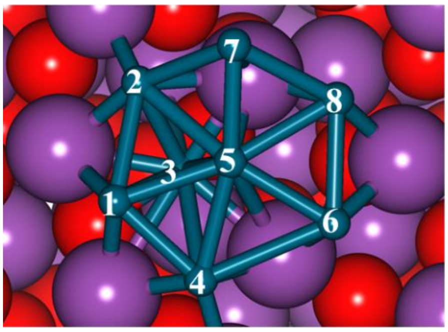

No. of Pd atom	Charge / e
1	-0.29
2	-0.39
3	-0.01
4	-0.37
5	-0.16
6	-0.30
7	-0.28
8	-0.36
	-0.27 (Average)

Supplementary Fig. 11 | Bader charge of Pd atoms on the Pd_8 cluster model. A negative charge here means the atom withdraws electrons from other atoms. Pd: cyan, Bi: purple, O: red.

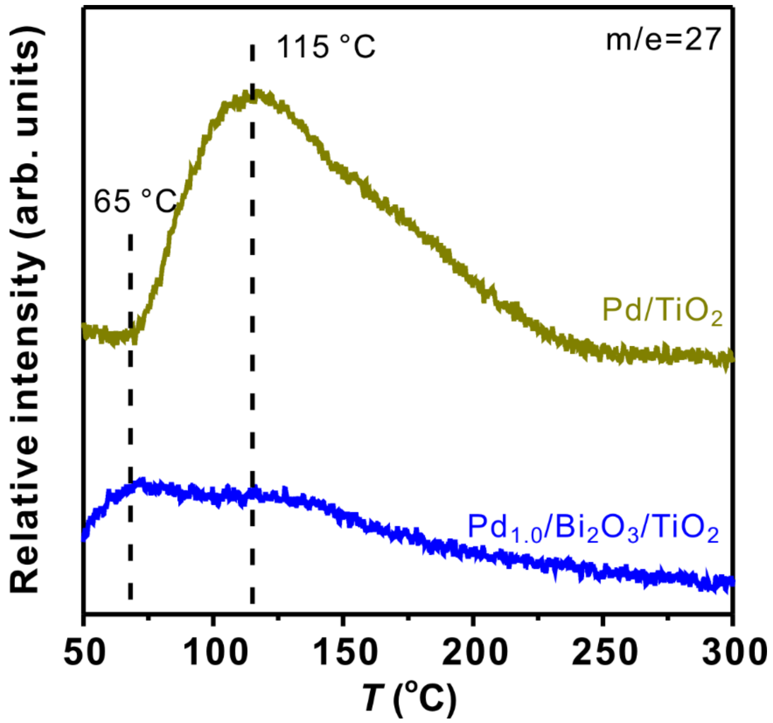

Supplementary Fig. 12 | C_2H_4 TPD profiles for Pd/TiO_2 and $Pd_{1.0}/Bi_2O_3/TiO_2$ (*m/e* = 27). Source data are provided in a Source Data file.

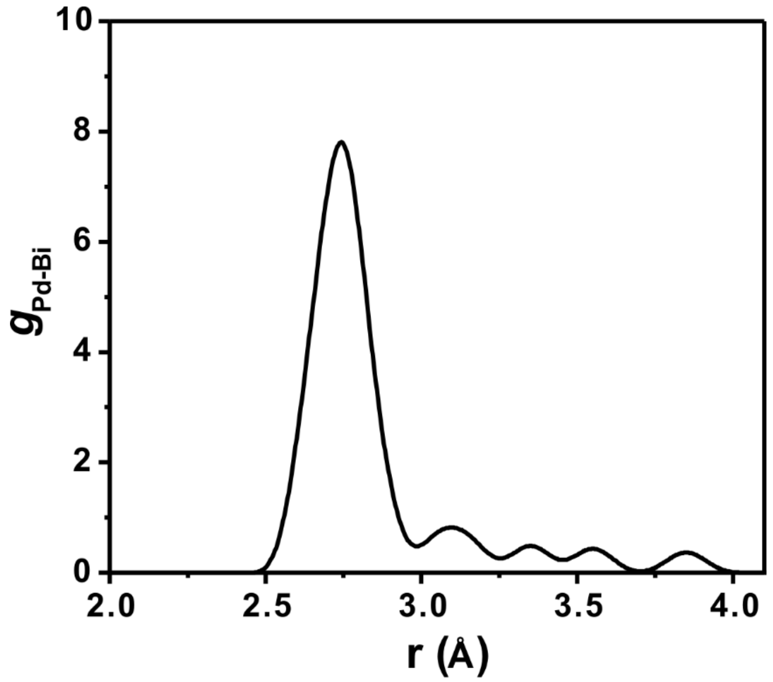

Supplementary Fig. 13 | Pd-Bi pair distribution function of the Pd_8 cluster structure. Source data are provided in a Source Data file.

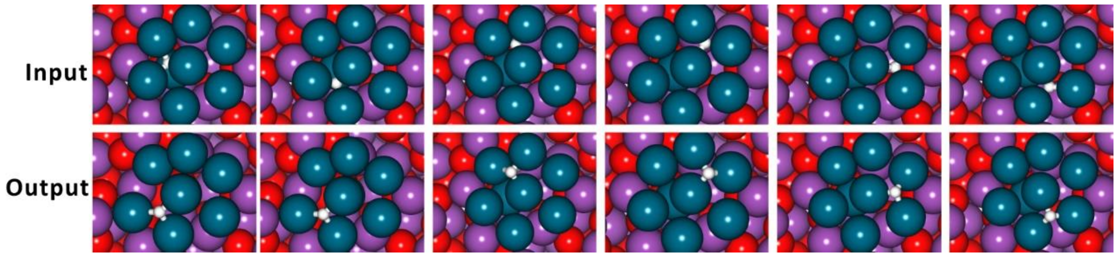

Supplementary Fig. 14 | Stability analysis of Pd-hydride. Input structures shows six configurations of subsurface H and they are considered as Pd-hydride; Output structures are obtained by optimizing the input structures, showing surface H. The results indicate that subsurface H is not stable and it transfers to surface during optimization. Pd: cyan, Bi: purple, O: red, H: white.

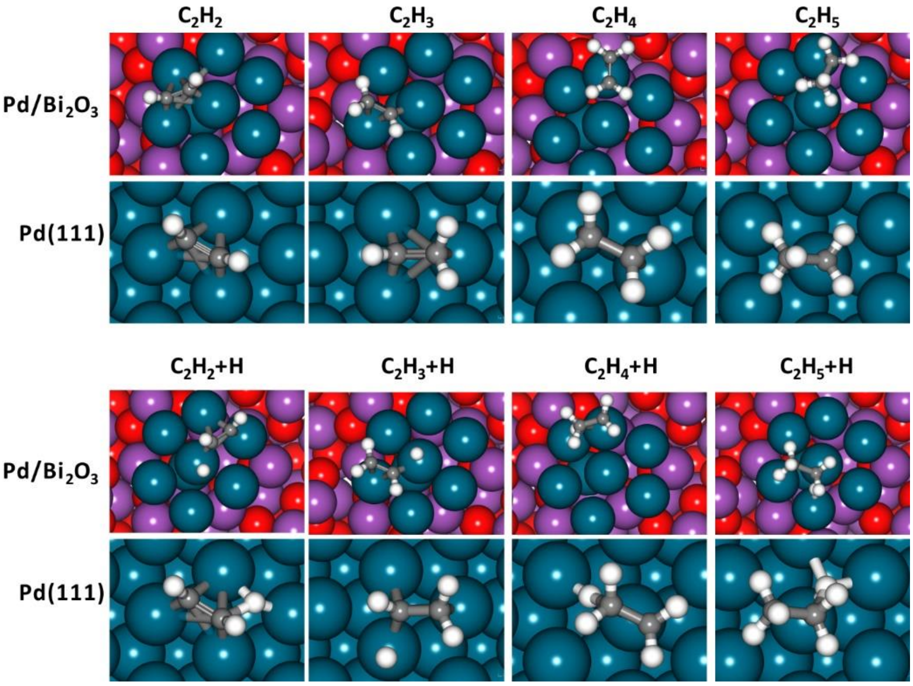

Supplementary Fig. 15 | Optimized structures for DFT calculations. Optimized structures of reactants, intermediates and transition states during C_2H_2 hydrogenation over Pd-cluster and Pd(111) structures. Pd: cyan, Bi: purple, O: red, C: grey, H: white.

Supplementary Table 3. DFT calculations of PdBi intermetallic from Crystallography Open Database (https://nanocrystallography.org/).

Composition and No. in the database	Pd-Bi CN	Pd-Bi distance /Å
PdBi No. 9004224	6	2.905
$PdBi_2$ No. 9012857	8	3.029
PdBi No. 9012856	7	2.893
$PdBi_2$ No. 9012855	7	2.846

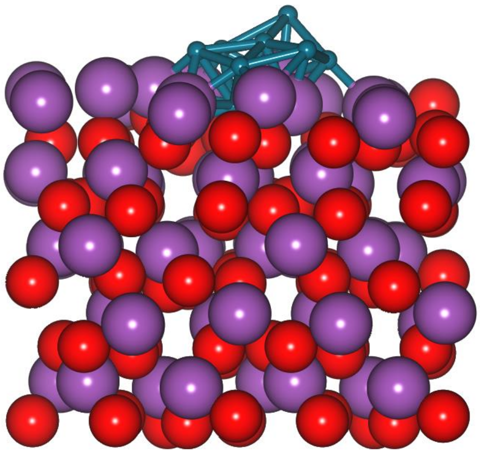

Supplementary Fig. 16 | Structure of the Pd_8-model with its bottom surface terminated by O. The number of Bi and O atoms follow the stoichiometric ratio of Bi_2O_3. Pd: cyan, Bi: purple, O: red.

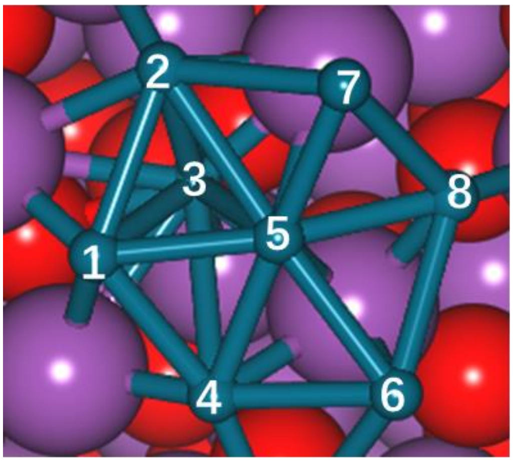

No. of Pd atom	Charge /e
1	-0.26
2	-0.26
3	-0.01
4	-0.32
5	-0.12
6	-0.24
7	-0.15
8	-0.32
	-0.21 (Average)

Supplementary Fig. 17 | Bader charge of Pd atoms on the Pd_8-cluster model with O-terminated bottom slab. A negative charge here means the atom withdraws electrons from other atoms. Pd: cyan, Bi: purple, O: red.

Supplementary Table 4. Activation energy (E_a) and reaction energy (ΔE) of the reactions involving CCH_2 and CCH_3 species on various catalyst surfaces. The unit of energy is in eV.

**Reaction**	***E_a / ΔE***		
	Pd-cluster in this work	Pt from literatures	Pd from literatures
$CHCH \rightarrow CHCH_2$	0.74 / -0.47	1.45 / -0.28[1]<br>~1.0 / ~-0.1[2]<br>0.85 / -0.03[3]<br>1.01 / -0.02[4]	0.96 / 0.19[5]<br>1.04 / 0.24[3]<br>0.96 /~0.2[6]
$CHCH \rightarrow CCH$	0.89 / -0.05	0.87 / 0.42[1]<br>1.48 / 0.82[3]<br>/ 1.02[7]<br>2.12 / 0.90[4]	1.23 / 0.25[5]<br>1.23 / 0.26[3]
$CHCH \rightarrow CCH_2$	2.98 / -1.10	/ -0.3[1]<br>/ -0.2[7]<br>/ -2.84[8]<br>2.60 / 0.22[4]	1.23 / -0.16[5]
$CHCH_2 \rightarrow CH_2CH_2$	0.53 / -0.72	1.15 / -0.21[1]<br>0.69 / ~-0.1[2]<br>0.70 / -0.13[3]<br>0.82 / -0.02[4]	0.97 / -0.05[5]<br>0.93 / -0.09[3]<br>0.89 / ~-0.3[6]
$CHCH_2 \rightarrow CHCH$	1.21 / 0.47	1.73 / 0.28[1]<br>0.90 / ~0.1[2]<br>1.03 / 0.02[4]	0.79 / -0.17[5]
$CHCH_2 \rightarrow CCH_2$	1.51 / -0.63	0.97 / -0.47[1]<br>/ -0.3[2]<br>0.70 / -0.20[4]	0.59 / -0.34[5]
$CHCH_2 \rightarrow CCH_3$	2.05 / -0.01	/ -0.3[1]<br>/ -0.48[8]<br>1.96 / -0.54[4]	0.80 / -0.70[5]

Supplementary Table 5. Energy barriers of acetylene hydrogenation to ethane over Pd(111). The unit of energy is in eV.[a]

Reaction	Barrier in this work /eV	Imaginary frequencies /cm^{-1}	Barrier in literature /eV
CHCH→$CHCH_2$	0.88	74.48	0.96[5,6]<br>0.55[9]<br>1.04[3]
$CHCH_2$→CH_2CH_2	0.85	97.10	0.89[6]<br>0.77[9]<br>0.97[5]<br>0.93[3]
CH_2CH_2→CH_2CH_3	0.88	79.88	0.91[6]<br>0.88[9]
CH_2CH_2→CH_3CH_3	0.65	119.00	0.57[9]

[a]The calculated barriers are generally consistent with reported values, demonstrating our calculated results are reliable. In addition, we further calculated the vibrational frequency based on the transition state structure during acetylene hydrogenation on Pd(111). All the transition states are characterized to possess only one imaginary frequency, further demonstrating the reliability of the constrained minimization method that we used in this work.

Supplementary Discussion

The principle of model development. Pd_8 cluster supporting on Bi-terminated Bi_2O_3 was built as the model for $Pd_{1.0}/Bi_2O_3/TiO_2$. We developed this model based on the idea that the model used in calculations should be consistent with experimental observations. i) The size of Pd cluster on $Bi_2O_3(100)$ is around 1.6 nm × 1.5 nm, which is the same as the particle size determined by HRTEM (Fig. 1). ii) The Pd_8 cluster model shows an average Pd-Pd coordination number (CN) of 4.0, which is close to the experimental values measured, i.e., 4.7 ± 0.5 (Supplementary Table 2). The obtained interatomic distance between Pd and Bi on Pd_8-cluster structure (~ 2.75 Å) is similar with experimental values (~ 2.79 Å). iii) The model is stable and would not deform under reaction condition.

The choice of Bi_2O_3(100). In this study, α-Bi_2O_3 (monoclinic, $P2_1/c(14)$, a= 5.849 Å, b= 8.166 Å and c= 7.510 Å) was formed by mild oxidation of Bi^0 in the air. We therefore used the structural parameters of α-Bi_2O_3 in DFT calculations. The optimized lattice parameters of Bi_2O_3 were a= 5.981 Å, b= 8.340 Å and c= 7.591 Å. We chose the (100) surface of Bi_2O_3 in the calculations because of several reasons. Firstly, (100) surface is a low-index surface that normally has large proportions among the overall crystal surfaces, which makes this surface representative. Secondly, the $Bi_2O_3(100)$ surface has a layered structure, and the Bi-terminated surface can be exposed under reducing conditions. We compared the stability between the stoichiometric Bi_2O_3 and oxygen-lean Bi_2O_3 under reducing conditions. The transition between the stoichiometric Bi_2O_3 ($Bi_xO_{1.5x}$) to oxygen-lean Bi_2O_3 (Bi_xO_y) structure can be regarded as the reaction:

$$Bi_xO_{1.5x} + (1.5x - y)H_2 \rightarrow Bi_xO_y + (1.5x - y)H_2O \quad (1)$$

And the free energy free of the reaction can be obtained by:

$$\Delta G = (1.5x - y)G_{H_2O} + G_{Bi_xO_y} - (1.5x - y)G_{H_2} - G_{Bi_xO_{1.5x}} \quad (2)$$

Using the above methods, we found it is spontaneous to reduce the O-terminated layer of stoichiometric $Bi_2O_3(100)$ surface with H_2. The result demonstrates the stability of the Bi-terminated $Bi_2O_3(100)$ surface under reducing conditions. Thirdly, we found that only such Bi-terminated $Bi_2O_3(100)$ surface can give rise to consistent Pd-Bi distances with those determined experimentally.

The Bi-termination of Bi_2O_3. Theoretically, O-termination is thermodynamically more stable than Bi-termination as it has a lower surface energy (0.0587 versus 0.0978 eV/Å^2). However, in reducing conditions, the Bi-termination is favored. We calculated the free energy change of removing surface oxygen atoms on O-terminated (100) surfaces with hydrogen to form a Bi-terminated surface. In the calculations, we utilized a 11-layer 1×1 Bi_2O_3(100) surface consisting of 44 Bi and 66 O atoms to represent an O-exposed surface. Then we removed the 6 surface O atoms to obtain the structure exposing surface Bi atoms. The free energy change (ΔG) can be calculated as follows:

$$\Delta G = (G_{\mathrm{Bi_{44}O_{60}}} + 6(G_{H_2O} - G_{H_2}) - G_{\mathrm{Bi_{44}O_{66}}})/N \qquad (3)$$

where the G of solid materials is equal to their corresponding DFT-energy (E). The free energies of H_2O and H_2 were calculated at 40 °C, which is close to the synthesis temperature of $Pd_{1.0}/Bi_2O_3/TiO_2$ and acetylene hydrogenation temperature. The entropy of gaseous species was obtained from NIST database (https://cccbdb.nist.gov/introx.asp). N is the number of O atoms that have been removed during the process and it is 6. It was found that the average ΔG is negative, i.e., −1.81 eV when removing the surface O atom, showing that the O atoms on the (100) surface are readily removed under reaction conditions, and the Bi-layer would be exposed. Therefore, we used the Bi-terminated Bi_2O_3(100) for further studies.

We used the slab without O-occupied bottom terminations to keep the surface layer identical to the bottom layer and to avoid possible effect of dipole moment on energy calculations. This treatment won't significantly affect the surface and electronic structure of Pd. To confirm this idea, we calculated the Bi_2O_3 structure with the O-terminated bottom slab. The structure is given in Supplementary Fig. 16, and the electron-transfer result is given in Supplementary Fig. 17**.** One can find from the figure that the charge-transfer effect of this structure follows the same trend as the Bi-terminated bottom slab (Supplementary Fig. 11), i.e., the Bi_2O_3 transfers electrons to Pd. Taking all the above discussion together, the Pd_8-cluster model used in the manuscript is reliable.

The effect of spectator species. In literatures, spectator species (CCH_2 and CCH_3) can be favorably formed on some Pt and Pd catalysts [1-8]. As shown in Supplementary Ref. 1[1], CHCH would be favorably dehydrogenated to form CCH on Pt catalyst, which is a precursor to for the formation of CCH_2. This reference also indicates that $CHCH_2$ can

be favorably dehydrogenated to CCH_2. In addition, the results from Supplementary Ref. 5[5] show that the dehydrogenation from $CHCH_2$ to CCH_2 on Pd catalyst is favorable.

In contrast, for the Pd-cluster structure studied in this work, it is found that the hydrogenation of CHCH to $CHCH_2$ has lowest activation energy (E_a) of 0.74 eV compared with the dehydrogenation or hydrogen shift of CHCH (Supplementary Table 3). This strongly suggests that formation of CCH_2 would be unfavorable on the Pd-cluster structure. Similarly, the hydrogenation of $CHCH_2$ to CH_2CH_2 has the lowest E_a among all reactions starting from $CHCH_2$, again indicating that the spectator species CCH_3 is hard to form on Pd-cluster model.

Based on the analysis above, CCH_2 and CCH_3 were indeed spectator species on some Pd and Pt catalysts. However, in our work the spectator species is hard to form on Pd-cluster structure. To this end, the effects of spectator species are not discussed.

Supplementary References

[1] Wang, Y., Dong, X., Yu, Y. & Zhang, M. Ethylene Decomposition over Pt(100): A Mechanism Study from First Principle Calculation. *Appl. Surf. Sci.* **390**, 984-992 (2016).

[2] Podkolzin, S. G., Alcalá, R. & Dumesic, J. A. Density Functional Theory Studies of Acetylene Hydrogenation on Clean, Vinylidene- and Ethylidyne-Covered Pt(111) Surfaces. *J. Mol. Catal. A* **218**, 217-227 (2004).

[3] Basaran, D., Aleksandrov, H. A., Chen, Z.-X., Zhao, Z.-J. & Rösch, N. Decomposition of Ethylene on Transition Metal Surfaces M(111). A Comparative DFT Study of Model Reactions for M=Pd, Pt, Rh, Ni. *J. Mol. Catal. A* **344**, 37-46 (2011).

[4] Chen, Y. & Vlachos, D. G. Hydrogenation of Ethylene and Dehydrogenation and Hydrogenolysis of Ethane on Pt(111) and Pt(211): A Density Functional Theory Study. *J. Phys. Chem. C* **114**, 4973-4982 (2010).

[5] Chen, Z.-X., Aleksandrov, H. A., Basaran, D. & Rösch, N. Transformations of Ethylene on the Pd(111) Surface: A Density Functional Study. *J. Phys. Chem. C* **114**, 17683-17692 (2010).

[6] Yang, B., Burch, R., Hardacre, C., Headdock, G. & Hu, P. Influence of Surface Structures, Subsurface Carbon and Hydrogen, and Surface Alloying on the Activity and Selectivity of Acetylene Hydrogenation on Pd Surfaces: A Density Functional Theory Study. *J. Catal.* **305**, 264-276 (2013).

[7] Gao, J., Zhao, H., Yang, X., Koel, B. E. & Podkolzin, S. G. Controlling Acetylene Adsorption and Reactions on Pt–Sn Catalytic Surfaces. *ACS Catal.* **3**, 1149-1153 (2013).

[8] Watwe, R. M., Spiewak, B. E., Cortright, R. D. & Dumesic, J. A. Density Functional Theory (DFT) Studies of C_1 and C_2 Hydrocarbons Species on Pt Clusters. *J. Catal.* **180**, 184-193 (1998).

[9] Studt, F., Abild-Pedersen, F., Bligaard, T., Sørensen, R. Z., Christensen, C. H. & Nørskov, J. K. Identification of Non-Precious Metal Alloy Catalysts for Selective Hydrogenation of Acetylene. *Science* **320**, 1320-1322 (2008).
